# DeepGRN: prediction of transcription factor binding site across cell-types using attention-based deep neural networks

**DOI:** 10.1186/s12859-020-03952-1

**Published:** 2021-02-01

**Authors:** Chen Chen, Jie Hou, Xiaowen Shi, Hua Yang, James A. Birchler, Jianlin Cheng

**Affiliations:** 1 Electrical Engineering and Computer Science Department, University of Missouri, Columbia, MO 65211 USA; 2 Department of Computer Science, Saint Louis University, St. Louis, MO 63103 USA; 3 Division of Biological Sciences, University of Missouri, Columbia, MO 65211 USA

**Keywords:** Transcription factor, Attention mechanism, DNA binding site prediction

## Abstract

**Background:**

Due to the complexity of the biological systems, the prediction of the potential DNA binding sites for transcription factors remains a difficult problem in computational biology. Genomic DNA sequences and experimental results from parallel sequencing provide available information about the affinity and accessibility of genome and are commonly used features in binding sites prediction. The attention mechanism in deep learning has shown its capability to learn long-range dependencies from sequential data, such as sentences and voices. Until now, no study has applied this approach in binding site inference from massively parallel sequencing data. The successful applications of attention mechanism in similar input contexts motivate us to build and test new methods that can accurately determine the binding sites of transcription factors.

**Results:**

In this study, we propose a novel tool (named DeepGRN) for transcription factors binding site prediction based on the combination of two components: single attention module and pairwise attention module. The performance of our methods is evaluated on the ENCODE-DREAM in vivo Transcription Factor Binding Site Prediction Challenge datasets. The results show that DeepGRN achieves higher unified scores in 6 of 13 targets than any of the top four methods in the DREAM challenge. We also demonstrate that the attention weights learned by the model are correlated with potential informative inputs, such as DNase-Seq coverage and motifs, which provide possible explanations for the predictive improvements in DeepGRN.

**Conclusions:**

DeepGRN can automatically and effectively predict transcription factor binding sites from DNA sequences and DNase-Seq coverage. Furthermore, the visualization techniques we developed for the attention modules help to interpret how critical patterns from different types of input features are recognized by our model.

## Background

Transcription factors (TFs) are proteins that bind to specific genomic sequences and affect numerous cellular processes. They regulate the rates of transcriptional activities of downstream genes through such binding events, thus acting as activators or repressors in the gene regulatory networks by controlling the expression level and the protein abundance of their targeted genes [1]. Chromatin immunoprecipitation-sequencing (ChIP-Seq) is the golden standard to determine the interactions of a TF and all its potential binding regions on genomic sequences. However, ChIP-Seq experiments usually require reagents and materials that are infeasible to acquire, such as antibodies targeting specific TF of interest. Thus, predictions of potential binding sites through computational methods are considered as alternative solutions. Also, the prediction of binding sites of TFs would facilitate many biological studies by providing resources as reference for experimental validation.

Many algorithms have been developed to infer the potential binding sites of different TFs, including hidden Markov models [2, 3], hierarchical mixture models [4], support vector machines [5, 6], discriminative maximum conditional likelihood [7] and random forest [8, 9]. These methods usually rely on prior knowledge about sequence preference, such as position weight matrix [10]. However, these features may be less reliable if they are generated from inference based methods (such as de-novo motif discovery) when no prior knowledge is available [7].

More recently, methods based on deep neural networks (DNNs), such as DeepBind, TFImpute, and DeepSEA, have shown performances superior to traditional models [11–13]. Compared with the conventional methods, deep learning models have their advantages at learning high-level features from data with huge sizes. This property makes them ideal for the binding site prediction task since a genome-wide binding profile of a TF can be generated from each ChIP-Seq experiment. Unlike many existing models that rely on the quality of the input data and labor-intensive feature engineering, deep learning requires less domain knowledge or data pre-processing and is more powerful when there is little or no prior knowledge of potential binding regions. Current studies in the protein binding site prediction tasks usually involve the combination of two deep learning architectures: convolutional neural networks (CNN) and recurrent neural networks (RNN). The convolutional layer has the potential to extract local features from different genomic signals and regions [14], while the recurrent layer is better at utilizing useful information across the entire sequences of data. Several popular methods for binding prediction, such as DanQ [15], DeeperBind [16], and FactorNet [17], are built on such model architecture.

Recently, the concept of attention mechanism has achieved great success in neural machine translation [18] and sentiment analysis [19]. It enhances the ability of DNNs by focusing on the information that is highly valuable to successful prediction. Combining with RNNs, it allows models to learn the high-level representations of input sequences with long-range dependencies. For example, long short-term memory (LSTM) models with attention mechanism have been proposed in relation classification [20] and sentence compression [21]. Because of the input context similarities between language processing (sentences) and the DNA binding site prediction (sequences and results from massively parallel sequencing), similar approaches can be applied improve the performance of existing methods [22–24].

Interrogating the input–output relationships for complex models is another important task in machine learning. The weights of a deep neural network are usually difficult to interpret directly due to their redundancy and nonlinear relationship with the output. Saliency maps and feature importance scores are conventional approaches for model interpretation in machine learning involving genomics data [25]. With the application of attention mechanism, we are also interested in testing its ability to enhance the interpretability of existing CNN-RNN architecture models.

In this paper, we develop a TF binding prediction tool (DeepGRN) that is based on deep learning with attention mechanism. The experimental results demonstrate that our approach is competitive among the current state-of-the-art methods. Also, our work can be extended to explain the input–output relationships through the learning process. We show that the utilization of informative patterns in both DNase-Seq and DNA sequences is important for accurate prediction.

## Implementation

### Datasets from ENCODE-DREAM challenge

The datasets used for model training and benchmarking are from the 2016 ENCODE-DREAM in vivo Transcription Factor Binding Site Prediction Challenge. The detailed description of the pre-processing of the data can be found at https://www.synapse.org/#!Synapse:syn6131484/.

For all TF and cell-types provided in the challenge datasets, the label of the binding status of the TFs is generated from ChIP-Seq experiments and used as ground truth. Chromatin accessibility information (DNase-Seq data), and RNA-Seq data are provided as input features for model training.

For model training, we follow the rules and restrictions of the DREAM challenge: the models are trained on all chromosomes except 1, 8, and 21, and chromosome 11 is used as validation. The model with the best performance in validation data is used for final prediction if no “leaderboard” dataset is provided by the challenge. The leaderboard data are available for some TFs for benchmarking, and each participant can test the performance on these TFs with up to ten submissions. Thus, if such data are provided, we pick the top 10 best models from the first step as an optional model selection step. The final performance of our models is reported based on the final test data that are used to determine the rank of the submissions in the challenge (Figure S1 and Table S1, see Additional file 1). We use the similar organization of input features introduced by FactorNet [17]: DNA Primary sequence, Chromatin accessibility information (DNase-Seq data) are transformed into sequential features and become the input of the convolution layers at the first part of the models. Gene expression and annotations are transformed into non-sequential features and feed into the intermediate dense layers of the model (Details are described in the “Deep neural network models with attention modules” section).

We also collected DNase and ChIP profiles for additional cell lines from the Encode Project (https://www.encodeproject.org) and Roadmap Epigenomics databases (http://www.roadmapepigenomics.org/data/) to improve the capability of generalization of our model. The performance of models trained with and without external datasets are evaluated separately.

### Transcription factor binding data

Transcription factor binding data from ChIP-Seq experiments is the target for our prediction. The whole genome is divided into bins of 200 bp with a sliding step size of 50 bp (i.e., 250-450 bp, 300-500 bp). Each bin falls into one of the three types: bound, unbound, or ambiguous, which is determined from the ChIP-Seq results. Bins overlapping with peaks and passing the Irreproducible Discovery Rate (IDR) check with a threshold of 5% [26] are labeled as bound. Bins that overlap with peaks but fail to pass the reproducibility threshold are labeled as ambiguous. All other bins are labeled as unbound. We do not use any ambiguous bins during the training or validation process according to the common practice. Therefore, each bin in the genomic sequence will either be a positive site (bounded) or a negative site (unbounded).

### DNA primary sequence

Human genome release hg19/GRCh37 is used as the reference genome. In concordance with the common practice of algorithms that perform feature extraction from chromatin profile, such as FactorNet[17], DeepSea[12], and DanQ[15], we expand each bin by 400 bp in both upstream and downstream, resulting in a 1000 bp input region. In addition, we have evaluated the performance of different selections of input ranges and showed that range above 600 bp is sufficient to acquire stable prediction performance (Figure S2). The sequence of this region is represented by a 1000 × 4 bit matrix by 1-hot encoding, with each row represented a nucleotide. Since low mappability sequences may introduce bias in parallel sequencing experiments, sequence uniqueness (also known as “mappability”) is closely related to the quality of sequencing data [27]. Thus, we select Duke 35 bp uniqueness score (https://genome.ucsc.edu/cgi-bin/hgFileUi?db=hg19&g=wgEncodeMapability) as an extra feature. Scores ranging from 0 to 1 are assigned to each position as the inverse of occurrences of a sequence with the exceptions that the scores of unique sequences are 1 and scores of sequences occurring more than four times are 0 [28]. As a result, the sequence uniqueness is represented by a 1000 × 1 vector for each input bin. The ENCODE Project Consortium has provided a blacklist of genomic regions that produce artifact signals in NGS experiments [29]. We exclude input bins overlapping with these regions from training data and set their prediction scores to 0 automatically if they are in target regions of prediction.

### DNase-Seq data

Chromatin accessibility refers to the accessibility of regions on a chromosome and is highly correlated with TF binding events [4]. DNase-Seq experiment can be used to obtain genome-wide maps of chromatin accessibility information as chromatin accessible regions are usually more sensitive to the endonuclease DNase-I than non-accessible regions [30]. DNase-Seq results for all cell-types are provided in the Challenge datasets in the BigWig format. Normalized 1 × coverage score is generated from the BAM files using deepTools [31] with bin size = 1 and is represented by a 1000 × 1 vector for each input bin.

### Gene expression and annotation

The annotation feature for each bin is encoded as a binary vector of length 6, with each value represent if there is an overlap between the input bin and each of the six genomic features (coding regions, intron, promoter, 5′/3′-UTR, and CpG island). We also include RNA-Seq data since they can be used to characterize the differences in gene expression levels among different cell-types. Principal Component Analysis (PCA) is performed on the Transcripts per Million (TPM) normalized counts from RNA-Seq data of all cell-types provided by the Challenge. The first eight principal components of a cell-type are used as expression scores for all inputs from that cell-type, generating a vector of length 8. The processed data files for these features are provided in the FactorNet Repository (https://github.com/uci-cbcl/FactorNet/tree/master/resources). These non-sequential features are fused into the first dense layer in the model.

### PhastCons genome conservation tracks

We use the 100-way PhastCons conservation tracks [32] as a feature for additional models. The PhastCons scores are represented as base-by-base conservation scores generated from multiple alignments of 99 vertebrates to the human genome. Conserved elements along the genome are recognized from phylogenetic models, and the conservation score for each base is computed as the probability that it locates in such conserved regions. For each input bin, the PhastCons scores are represented as a vector of L × 1 with a range from 0 to 1.

### CpG island feature profiling

We use the CGI score derived from Mocap [33] to profile the epigenomic environment for each input region. The CGI score can be calculated as:

$$CGI\left( {N_{CpG} ,N_{C} ,N_{G} ,L} \right) = \left\{ {\begin{array}{*{20}c} {1\ if \frac{{N_{CpG} L}}{{((N_{C} + N_{G} )/2)^{2} }} > 0.6\ and\ \frac{{N_{C} + N_{G} }}{L} > 0.5} \\ {0 \ otherwise } \\ \end{array} } \right.$$

For each input bin, the CGI scores are represented as a vector of L × 1 with binary values of 0 or 1.

### Deep neural network models with attention modules

**Fig. 1:**
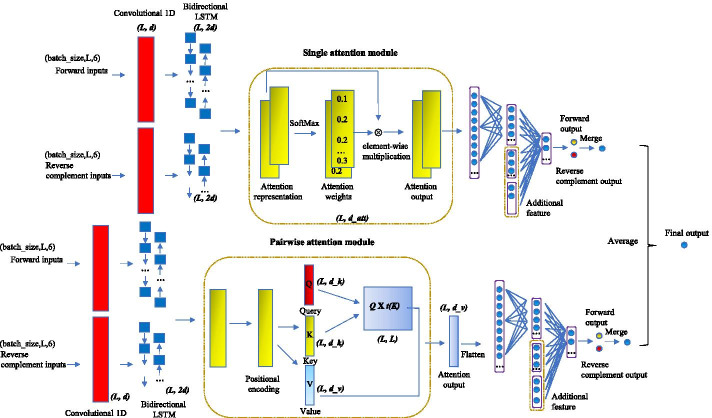
The general framework of the two attention modules of DeepGRN. The diagram of the deep neural network architecture. Convolutional and bidirectional LSTM layers use both forward and reverse complement features as inputs. In the single attention module, attention weights are computed from hidden outputs of LSTM and are used to generate the weighted representation through an element-wise multiplication. In the pairwise attention module, three components: Q(query), K(key), and V(value) are computed from LSTM output. The multiplication of Q and transpose of K are used to calculate the attention weights for each position of V. The multiplication of V and attention scores is the output of the pairwise attention module. Outputs from attention layers are flattened and fused with non-sequential features (genomic annotation and gene expression). The final score is computed through dense layers with sigmoid activation and merging of both forward and reverse complement inputs. The dimensions of each layer are shown beside each component

The shape of each sequential input is L × (4 + 1 + 1) for each region with length L after combining all sequential features (DNA sequence, sequence uniqueness, and Chromatin accessibility). Sequential inputs are generated for both the forward strand and the reverse complement strand. The weights in all layers of the model are shared between both inputs to form a “Siamese” architecture [17, 34, 35]. Vectors of non-sequential features from gene expression data and genomic annotations are fused into the model at the first dense layer. The overall architecture of our model is shown in Fig. 1. The model is built with two major modules: single attention and pairwise attention. They use the same input and architecture except for their internal attention mechanism. The final result of our model is the average of the output of two modules.

The first part of our model is a 1D convolutional layer, which is a common practice for feature extraction in deep learning models involving genomics data [13, 17]. We use Bidirectional Long Short-term Memory (Bi-LSTM) nodes as recurrent units in our model. The computation steps in an LSTM unit can be written as:

1 $$f_{t} = \sigma \left( {W_{f} \cdot \left[ {h_{t - 1} ,x_{t} } \right] + b_{f} } \right)$$

2 $$i_{t} = \sigma \left( {W_{i} \cdot \left[ {h_{t - 1} ,x_{t} } \right] + b_{i} } \right)$$

3 $$\widetilde{{C_{t} }} = tanh\left( {W_{C} \cdot \left[ {h_{t - 1} ,x_{t} } \right] + b_{C} } \right)$$

4 $$\widetilde{{C_{t} }} = f_{t} * C_{t - 1} + i_{t} * \widetilde{{C_{t} }}$$

5 $$o_{t} = \sigma \left( {W_{o} \cdot \left[ {h_{t - 1} ,x_{t} } \right] + b_{o} } \right)$$

6 $$h_{t} = o_{t} * tanh\left( {\widetilde{{C_{t} }}} \right)$$

where $f_{t}$, $i_{t}$, and $o_{t}$ are the forget gate, input gate, and output gate. $h_{t - 1}$ and $h_{t}$ are the hidden state vectors at position $t - 1$ and $t$. $x_{t}$ is the input vector at position $t$. $\left[ {h_{t - 1} ,x_{t} } \right]$ stands for vector concatenation operation. $C_{t - 1}$, $\widetilde{{C_{t} }}$ and $C_{t}$ are output cell state at position $t - 1$, new cell state at position *t,* and output cell state at position $t$, respectively. $W_{f}$, $W_{i}$, $W_{C}$, and $W_{o}$ are learned weight matrices. $b_{f}$, $b_{i}$, $b_{C}$, and $b_{o}$ are learned bias vector parameters for each gate. $\sigma$ and $tanh$ are sigmoid function and hyperbolic tangent function, respectively.

In Bi-LSTM layers, two copies of the inputs of LSTM are rearranged into two directions: one for the forward direction and one for the backward direction, and they go into the LSTM unit separately. The outputs from two directions are concatenated at the last dimension. Thus, the last dimension of the Bi-LSTM output is two times of the last dimension of the input.

In the single attention module, suppose its input vector $h$ has shape $l$ by $r$, we first computed the unnormalized attention score $e = M \times h{ }$ where $M$ is a weight matrix with shape $l$ by $l$, and $e$ has shape $l$ by $r$. A learned bias of shape $l$ by $r$ is added to $e$ after the multiplication. This can be summarized as a dense layer operation $f_{att,r}$ on input $h$. Then, we apply the Softmax function along the first dimension of $e$ in order to get the normalized attention score $\alpha$. Finally, the weighted output $Z$ will be computed based on the attention weight $\alpha$. At dimension $r$ of input $h$, these steps can be written as follows:

7 $$e_{r} = f_{att,r} \left( {h_{1,r} ,h_{2,r} ,...,h_{N,r} } \right)$$

8 $${ }\alpha_{i,r} = exp\left( {e_{i,r} } \right)/\mathop \sum \limits_{k = 1}^{N} exp\left( {e_{k,r} } \right){ }{\text{ }}$$

9 $$\alpha_{i} = (\mathop \sum \limits_{r = 1}^{R} \alpha_{i,r} )/D$$

10 $$z_{i,r} = h_{i,r} {*}\alpha_{i}$$

Here, $e_{r}$ is the unnormalized attention score at dimension $r$. Vector $\alpha_{i,r}$ represents attention weight at dimension $r$ of position $i$ and is normalized by Softmax function. The attention dimension $r$ in our model will stay unchanged during the transformations. The dimension of the attention weights can be reduced from $N \times r$ to $N \times 1$ by averaging at each position. The final output $z_{i,r}$ is computed based on the corresponding attention score. After the attention layers, the prediction scores are computed from dense layers with sigmoid activation function and merged from both forward and reverse complement inputs.

In the pairwise attention module, there are three components: Q(query), K(key) and V(value). Their values are computed from LSTM output from three different trainable weight matrices. The dimension of the trained weights for Q, K and V are $l$ by $d_{k}$, $l$ by $d_{k}$ and $l$ by $d_{v}$ where $d_{k}$ and $d_{v}$ are set as 64 as the default setup described in [36]. The multiplication of Q and transpose of K are used to compute the attention weights for each position of V after Softmax conversion and dimension normalization. The multiplication of V and attention weights are the output of the pairwise attention module. The output of the pairwise attention module is computed as:

11 $$Z = Softmax\left( {\frac{{Q \times K^{T} }}{{\sqrt {d_{k} } }}} \right) \times V$$

Since each position in the sequential features simultaneously flows through the pairwise attention module, the pairwise attention module itself is not able to sense the position and order from the sequential input. To address this, we add the positional encodings to the input of the pairwise attention. We expect this additional encoding will enhance the ability of the model to make use of the order of the sequence. The positional encodings have the same dimension $d$ as the input of the pairwise attention module. In this work, we choose different frequencies sine and cosine functions [37] to encode the positional information:

12 $$PE_{{\left( {pos,2i} \right)}} = sin\left( {pos/10000^{2i/d} } \right)$$

13 $$PE_{{\left( {pos,2i + 1} \right)}} = cos\left( {pos/10000^{2i/d} } \right)$$

where $pos$ is the position in the sequential input, and $i$ is the index of the last dimension of the model. The resulting positional encodings vector is added to its input. Through such encoding technique, the relative position information can be learned by the model since for any fixed offset $k$, $PE_{{\left( {pos + k} \right)}}$ can be represented as $PE_{{\left( {pos,2i} \right)}} cos\left( {10000^{2k/d} } \right) + PE_{{\left( {pos,2i + 1} \right)}} sin\left( {10000^{2k/d} } \right)$, which is the linear combination of $PE_{{\left( {pos} \right)}}$. Similarly, this also applies to dimensions of $2i + 1$ as well.

The single attention module is designed to represent the importance of different regions along with the sequential input, while the pairwise attention module seeks to attend the importance between each pair of positions across the sequential input. We expect this difference in architecture will help to improve the learning ability of the model in a complementary manner.

We tested different configurations for typical hyperparameters (learning rate, network depth, dropout rates) and the hyperparameters specific to our model (the dimension of attention weights, merging function the two output scores) during training. The complete description of hyperparameters and their possible options are summarized in Table S2 [see Additional file 1]. We train one model for each TF, resulting in 12 models in total. The single and pairwise attention module will always use the same configuration rather than train separately.

There are 51,676,736 bins in total on training chromosomes in the labels, resulting in $51676736 \times n$ potential training samples for each TF, where $n$ is the number of available cell-types for training. Due to limited computing capacity, we use the iterative training process. During training, the training data is the mixture of all positives (labeled as “B”) with downsampled negatives (labeled as “U”) [17]. In the traditional model training in deep learning, all input data are used to update the model weights exactly once for each epoch. However, this is not applicable in our task since the negative samples (regions do not bind to TFs) are much more abundant than the positive samples (regions bind to TFs), and use all negative samples for training in one epoch is not practical since the number of them is extremely huge (as they cover most of the human genome). Thus, in each epoch during model training, we first sample negative samples with numbers proportional to the number of all positive samples, and combine these negative samples with all positive samples for training. We will re-sample the negative bins and start another round of model training (next epoch). To make the training process more effective, we use a different strategy to generate positive training samples for transcription factors that have a large number of positive labels (CTCF, FOXA1, HNF4A, MAX, REST and JUND). For these TFs, we randomly sample a 200-bp region from each ChIP-Seq peak in the narrow peak data as positive instances for training instead of using all positive samples in each epoch. We use the Adam [38] optimizer with binary cross-entropy as the loss function. The default number of epochs is set to 60, but the training will be early stopped if there are no improvements in validation auPRC for five consecutive epochs. For detailed instructions about data retrieving, training, prediction, and visualization with our programs, please see Additional file 2.

## Results

### Overall benchmarking on evaluation data

We list the performance of our model as four metrics used in the DREAM Challenge (Table 1) and compare them with the unified score from the top four teams in the final leaderboard of the ENCODE-DREAM Challenge (Table 2). The unified score for each TF and cell-type is based on the rank of each metric and is computed as: $\sum ln\left( {r/\left( 6 \right)} \right){ }$ where $r$ is the rank of the method for one specific performance measure (auROC, auPRC, Recall at 50% FDR and Recall at 10% FDR). Thus, smaller scores indicate better performance. The TFs, chromosomes, and cell-types for evaluation are the same as those used for the final rankings. DeepGRN typically achieves auROC scores above 98% for most of the TF/cell type pairs, reaching as low.

**Table 1:** The performance of DeepGRN with four metrics used in the DREAM Challenge

TF name	Cell-type	auROC	auPRC	Recall at 50% FDR	Recall at 10% FDR
CTCF	PC-3	0.987	0.767	0.766	0.603
CTCF	induced pluripotent stem cell	0.998	0.902	0.945	0.744
E2F1	K562	0.989	0.404	0.388	0.100
EGR1	liver	0.993	0.405	0.318	0.021
FOXA1	liver	0.985	0.546	0.584	0.164
FOXA2	liver	0.984	0.548	0.588	0.143
GABPA	liver	0.991	0.516	0.488	0.154
HNF4A	liver	0.971	0.636	0.700	0.263
JUND	liver	0.983	0.535	0.585	0.027
MAX	liver	0.990	0.425	0.349	0.004
NANOG	induced pluripotent stem cell	0.996	0.499	0.515	0.035
REST	liver	0.986	0.482	0.527	0.030
TAF1	liver	0.989	0.424	0.393	0.000

**Table 2:** The unified scores of DeepGRN and the top four algorithms in the DREAM Challenge

TF	Cell	Anchor	FactorNet	Cheburashka	Catchitt	DeepGRN
CTCF	PC-3	0.67	0.17	0.83	0.5	0.33
CTCF	induced pluripotent stem cell	0.83	0.33	0.67	0.5	**0.17**
E2F1	K562	0.5	0.83	0.67	0.17	0.33
EGR1	liver	0.17	0.83	0.67	0.33	0.5
FOXA1	liver	0.67	0.33	0.83	0.5	**0.17**
FOXA2	liver	0.33	0.83	0.67	0.5	**0.17**
GABPA	liver	0.33	0.83	0.67	0.5	**0.17**
HNF4A	liver	0.67	0.33	0.83	0.5	**0.17**
JUND	liver	0.17	0.83	0.67	0.5	0.33
MAX	liver	0.17	0.83	0.33	0.67	0.5
NANOG	induced pluripotent stem cell	0.33	0.5	0.83	0.67	**0.17**
REST	liver	0.67	0.33	0.83	0.5	**0.17**
TAF1	liver	0.17	0.5	0.67	0.33	0.83

Bold scores denote the TF and cell-types that DeepGRN rank as the highest

as 97.1% for HNF4A/liver. The scores of auPRC have a more extensive range of values, from 40.4% for E2F1/ K562 to 90.2% for CTCF/iPSC.

For each TF and cell-type combination, our attention model has better performance on 69% (9/13) of the prediction targets than Anchor [39], 85% (11/13) than FactorNet [17], 85% (11/13) than Cheburashka [7], and 77% (10/13) than Catchitt [40]. Among all methods benchmarked, our method has the highest ranking in 7 out of 13 targets (CTCF/iPSC, FOXA1/liver, FOXA2/liver, GABPA/liver, HNF4A/liver, NANOG/iPSC, and REST/liver), with the best average score (0.31) across all TF/ cell-types pairs (Table 2).

**Fig. 2:**
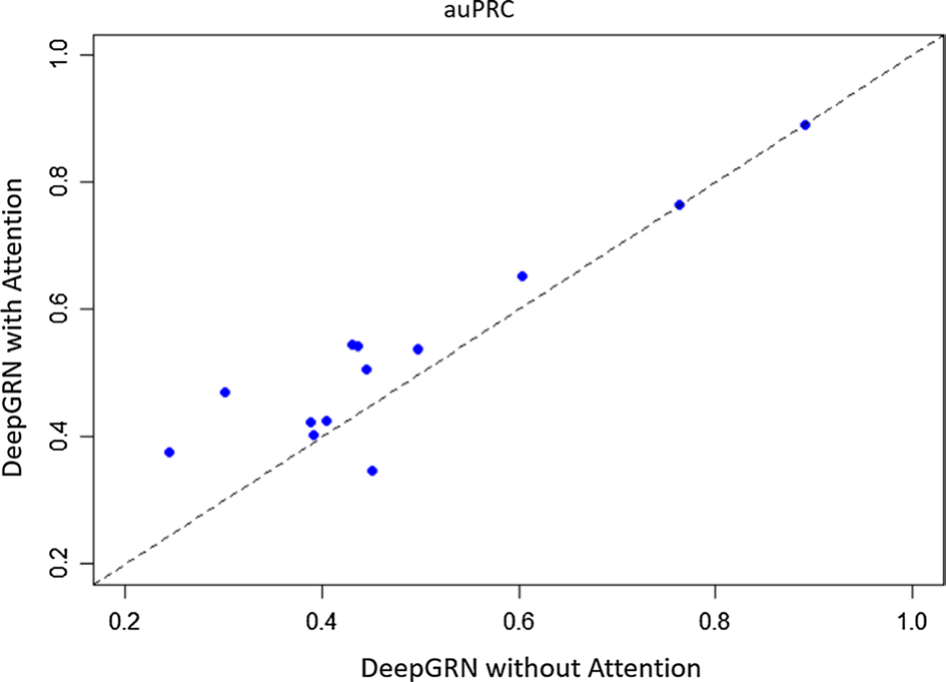
Comparision of the deep learning models with and without attention mechanism

To precisely evaluate the capability of deepGRN under the restrictions of the ENCODE DREAM Challenge, we also compared the performance of deepGRN trained using datasets provided by the challenge with four available features: Genomic sequence features, DNase-Seq and RNA-Seq data. The results are summarized in Table S3 and S4. DeepGRN still achieves the highest ranking in 6 out of 13 targets, with the best average unified score (0.33) across all targets. We also compared our results with models without the attention component using the four challenge features. We built these models using the same architecture as deepGRN models, except for the attention component and trained them with the same hyperparameter selection process. The results are shown in Fig. 2. DeepGRN with attention mechanism outperforms the models without attention in 11 out of 13 targets by the auPRC metric, with the largest difference from target REST (0.168).

### Performance comparison between two attention modules

**Fig. 3:**
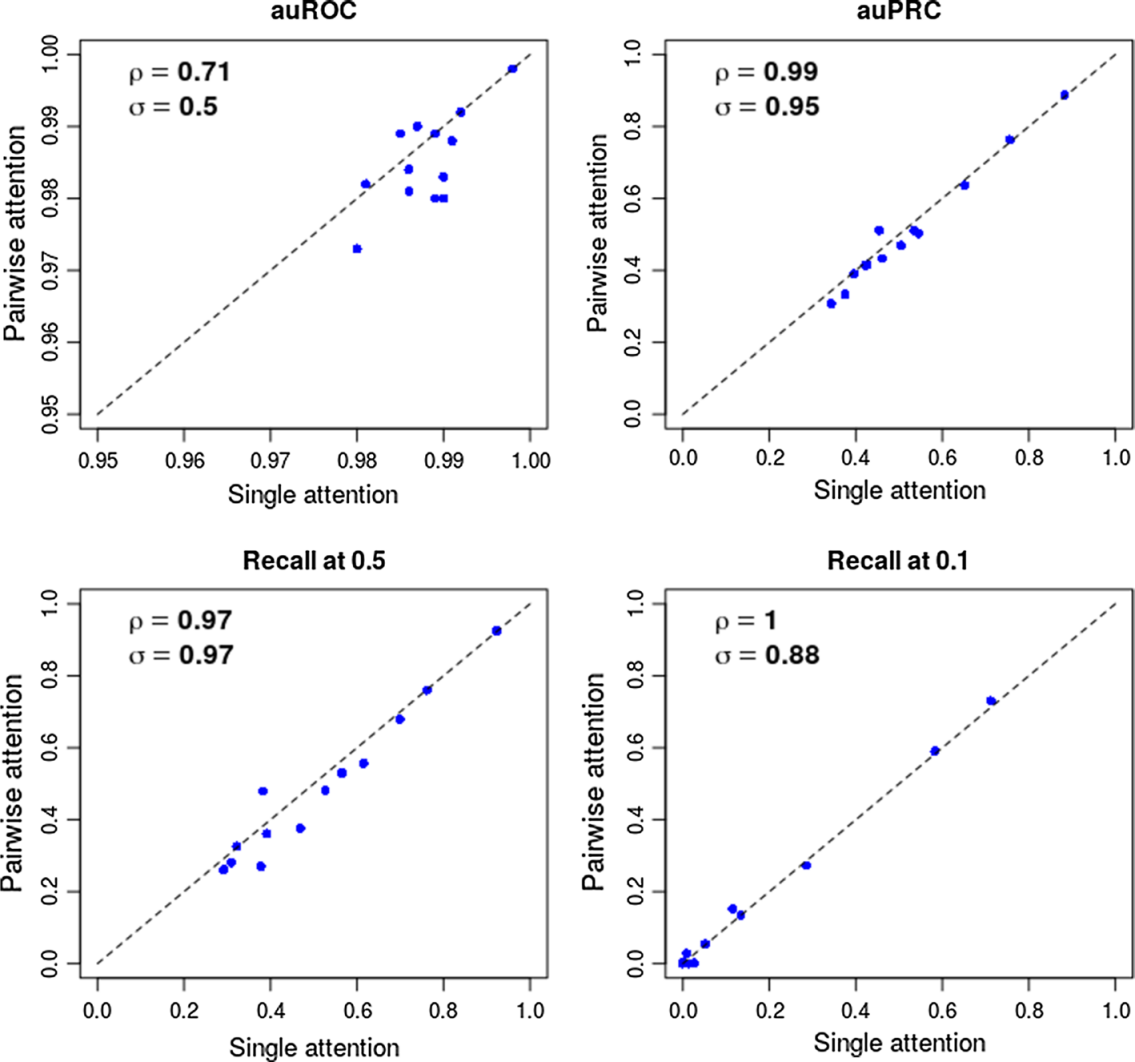
Performance comparison between single and pairwise attention mechanism. The performance of each TF and cell-type pairs of the output of the individual module are shown in four measures: (auROC, auPRC, recall at 50% FDR and Recall at 10% FDR). ρ: Pearson Correlation Coefficient, σ: Spearman Correlation Coefficient

In addition to the comparisons with the top 4 methods in the challenge, we also benchmarked the individual performance of the single and pairwise attention module (Table S5, see Additional file 1). In general, the results extracted from the single attention module have similar performances. For all 13 TF and cell-type pairs, the single attention module has higher auROC in 6 targets while the pairwise attention module has higher auROC in 3 targets. The rest of the targets are tied. The final output of the model is the ensemble of these two modules by averaging, and it outperforms any of the individual attention modules in 10 of 13 targets (Table 1). The largest improvements from ensemble (as auPRC) come from FOXA2 (0.34), REST (0.09) and FOXA1 (0.09). We also found that the performance of the two attention modules have the same trend across all TF and cell-types in all four performance measures (Fig. 3), suggesting that the capability of learning from features are coherent between the two modules.

**Fig. 4:**
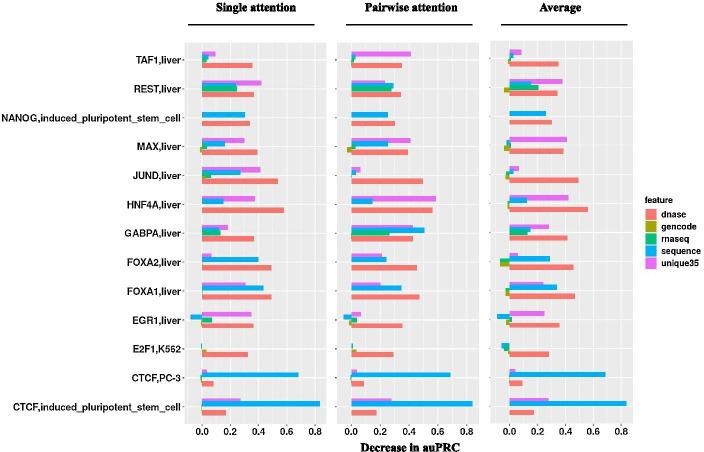
Importance score of features between single and pairwise attention mechanism. The values represented as the decrease of auPRC without using the specific feature for prediction. The negative value represents an increase of auPRC

We evaluated the importance of each feature between single and pairwise attention mechanism. For the prediction of each target, we set the values of each sequential feature (DNase-Seq, sequence, or uniqueness) to zero, or randomly switch the order of the vector for a non-sequential feature (genomic elements or RNA-Seq). The decrease of auPRC from these new predictions is used as the importance score of each feature (Fig. 4). We found that across all TF and cell-types, the sequential features have the largest average importance scores: DNase-Seq (0.36), DNA sequence (0.21), and 35 bp uniqueness (0.21) while the scores for other features are much smaller. Similar trends have also been found using individual single and pair attention modules.

### Interpretation of attention scores with DNase-Seq and ChIP-Seq

**Fig. 5:**
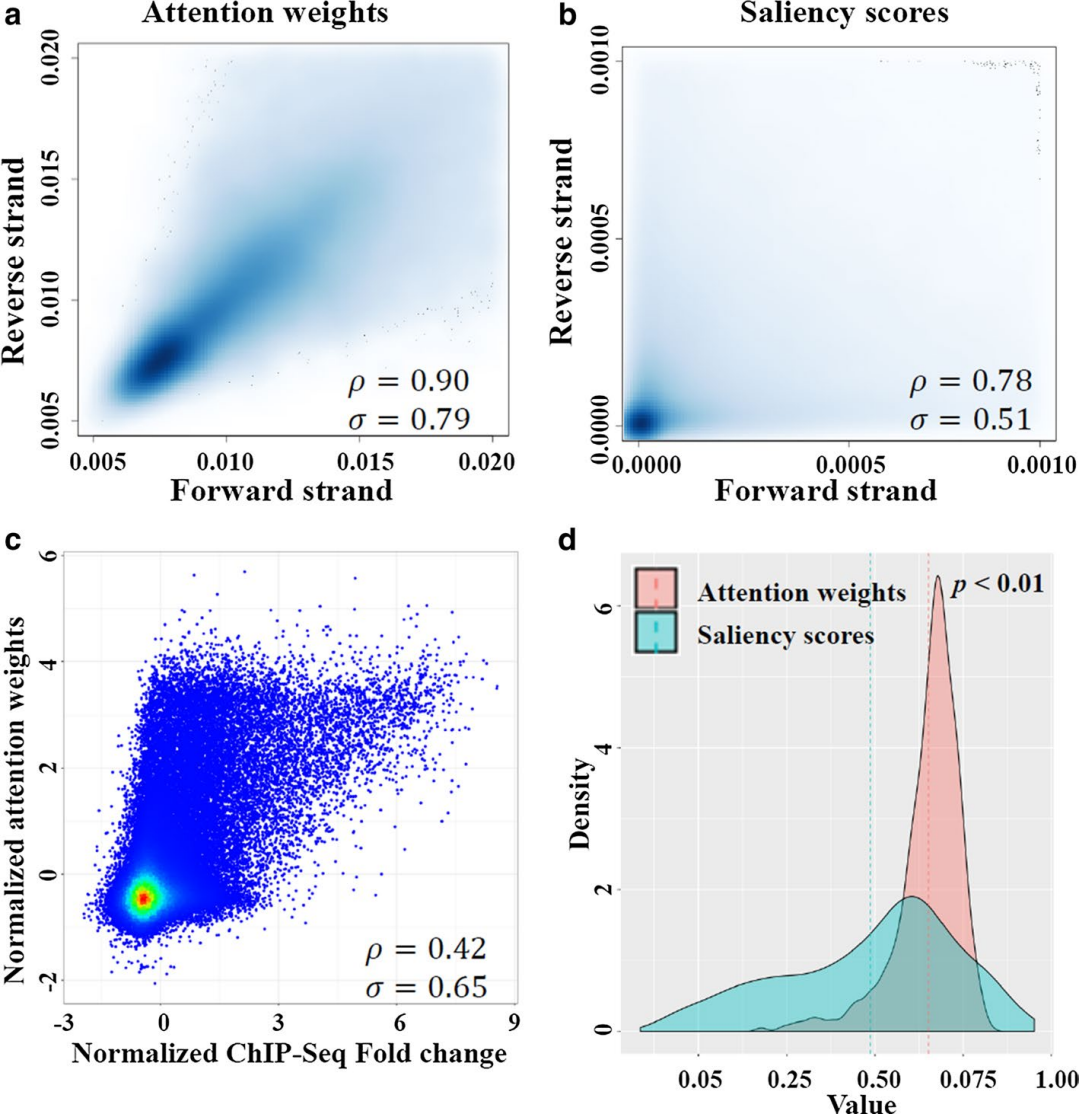
Analysis of attention weights and saliency scores. (**a**) Scatterplot of attention weights from positive strand and reverse strand. (**b**) Scatterplot of saliency scores from positive strand and reverse strand. (**c**) Scatterplot of ChIP-Seq fold change and mean attention weights from both strands. Z-score transformation is applied to both axes. (**d**) Distribution of the correlation between attention weights/saliency scores and ChIP-Seq fold change. The dashed line represents the mean of each group. The p-value is calculated using the Wilcoxon signed-rank test. The attention weights and saliency scores on the reverse complement strand are reversed before plotting. ρ: Spearman Correlation Coefficient, σ: Pearson Correlation Coefficient. The correlation between normalized ChIP-Seq Fold change and normalized saliency scores is 0.40 (Spearman) and 0.49 (Pearson)

In the single attention module, the output is a weighted sum of the input from the attention layer, and the attention scores are used as weights. These scores characterize a unified mapping between the importance of input feature with its relative position in the sequential input. To analyze the relationship between attention weights and the position of TF binding events, we extract the attention scores from the single attention module for both forward strand and reverse complement strand and compare them with the corresponding normalized ChIP-Seq fold changes in the same region that are predicted as positive (score > 0.5). Similarly, we computed the saliency scores for the same input regions (The implementation details are described in Additional file 1). We found that the attention scores on the two DNA strands have a higher correlation (ρ = 0.90, σ = 0.79) than the saliency scores (ρ = 0.78, σ = 0.51) (Fig. 5a, b). Across all TF and cell-type pairs, we found that there is a positive correlation between the attention weights and normalized ChIP-Seq Fold (Fig. 5c), and such relationship is not detected globally in saliency scores (Fig. 5d). For all TF and cell-types in the benchmark datasets, we select at least four different genomic regions that have a clear ChIP-Seq peak signal in each target for demonstration. We show that the averaged attention weights put more focus on the actual binding region for each cell-type and these focusing points shift along with the shift of TF binding signals (see Additional file 3).

**Fig. 6:**
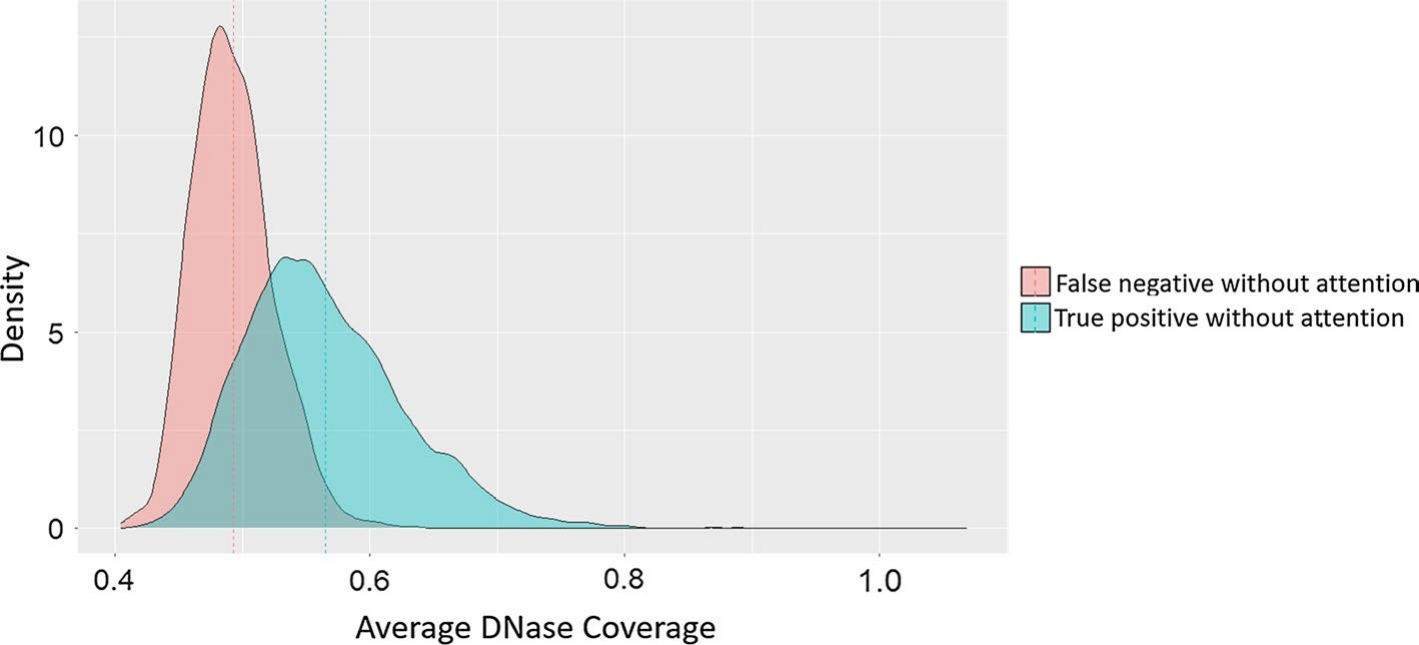
Distribution of average normalized DNase coverage values of different regions with the inputs of JUND. The predictions from both models with and without attention from our training are evaluated by the true positive labels. Then the average normalized DNase coverage is calculated based on bins classified differently by the two models

Since the accessibility of the genome plays an important role in TF binding, it is expected to find high DNase coverage for those openly accessible areas that can explain the binding event detected by the ChIP-Seq experiment. We run a genome-wide analysis on regions with high DNase-Seq peaks in the single attention module for transcription factor JUND, which is one of the most susceptible targets to DNase-Seq. We illustrate the distribution of normalized DNase coverage values from both the true positives that are false negatives without attention and true negatives that are false positives without attention (Fig. 6). The results show that the true positives that are only recognized by attention models generally have a smaller DNase coverage than those recognized by both models. This observation indicates that the predictive improvements of attention models may result from focusing on more informative DNase-Seq coverage values while ignoring irrelevant regions in negative samples.

### Motif detection over high attention scores regions

**Fig. 7:**
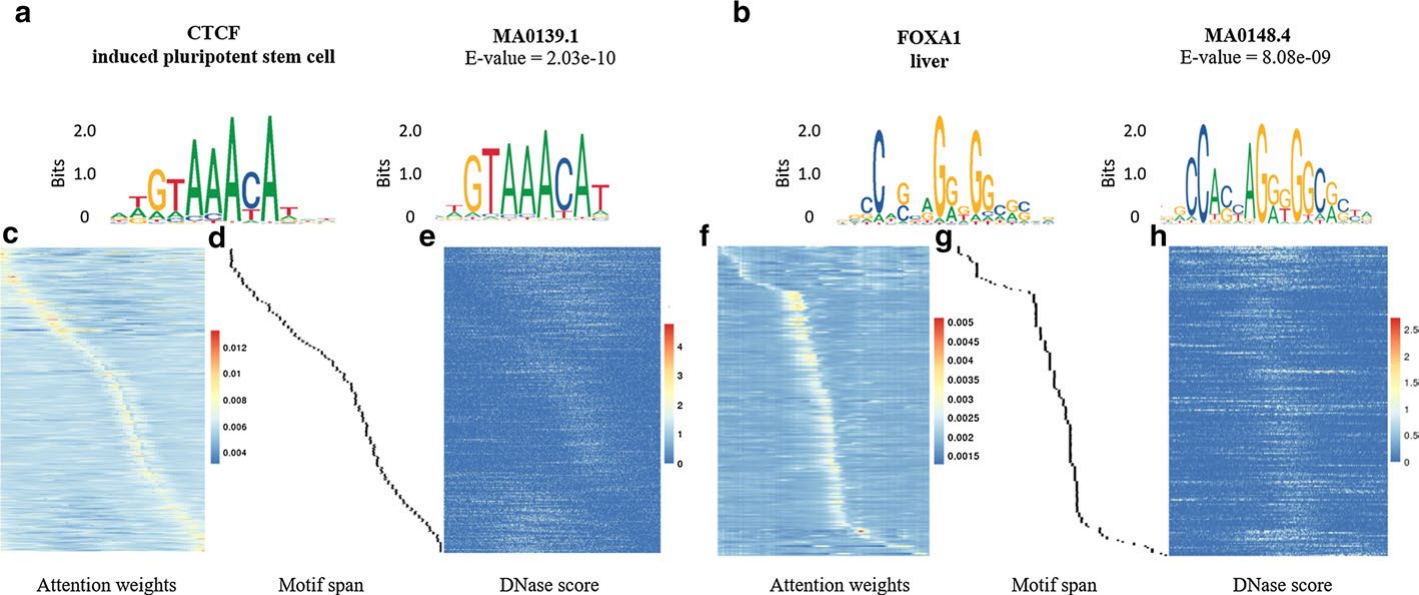
Comparisons of known motifs and matching motifs learned by pairwise attention module in CTCF and FOXA1. (**a**) Sequence logo built from subsequences detected in CTCF/induced pluripotent cell prediction (left) and motif MA0139.1/ CTCF (right). (**b**) The attention scores of the samples selected from CTCF/induced pluripotent cell prediction with hits of MA0139.1/ CTCF in FIMO. (**c**)The relative positions of the detected motifs in the same region of (**b**). (**d**) The normalized DNase-Seq scores in the same region of (**b**). (**e**) Sequence logo built from subsequences detected in FOXA1/liver cell prediction (left) and motif MA0148.4/ FOXA1 (right). (**f**) The attention scores of the samples selected from FOXA1/liver cell prediction with hits of MA0148.4/ FOXA1 in FIMO. (**g**) The relative positions of the detected motifs in the same region of (**f**). (**h**) The normalized DNase-Seq scores in the same region of (f)

For those positive samples without distinct DNase-Seq peaks, the patterns of genomic sequences are critical information for successful prediction. To test the ability of attention weights to recognize motifs that contribute to binding events from the genomic sequences, we use an approach similar to DeepBind [13]. For the model trained for each TF, we first acquire the coordinates on the relative positions of maximum column sum of the attention weights from all positive bins in test datasets and extract a subsequence with a length of 20 bp around each coordinate. To exclude samples that can be easily classified from patterns of DNase-Seq signal, we only select positive bins that have no significant coverage peaks (ratio between the highest score and average scores < 15). Then we run FIMO [41] to detect known motifs relevant to the TF of the model in the JASPAR database [42]. From the extracted subsequences, we discover motif MA0139.1 (CTCF) in the prediction for CTCF/induced pluripotent cell and MA0148.4 (FOXA1) in the prediction for FOXA1/liver cell. Figure 7a and b show the comparison between the sequence logo of the motif rebuilt from the subsequences and the actual known motifs. We also plot the attention scores of the samples that contain these subsequences (Fig. 7c, f) and the relative location of the regions with detected motifs in FIMO (Fig. 7d, g). Furthermore, we show that these maximum attention weights do not come from the DNase-Seq peaks near the motif regions by coincidence since no similar pattern is detected from the normalized DNase scores in the same regions (Fig. 7e, h). We illustrate the similar trends found in the single attention module in Figure S3 [see Additional file 1].

## Discussion

The attention mechanism is attractive in various machine learning studies and has achieved superior performance in image caption generation and natural language processing tasks [37, 43]. Recurrent neural network models with attention mechanism are particularly good at tasks with long-range dependency in input data. Inspired by these works, we introduce the attention mechanism to DNN models for TF binding site prediction.

The benchmark result using ENCODE-DREAM Challenge datasets shows that the performances of our model are competitive with the current state-of-the-art methods. It is worth mentioning that the DNase-Seq scores are the most critical feature in the attention mechanism from our experiments according to the feature importance analysis. Many prediction tools for binding site prediction before the challenge, such as DeepBind or TFImpute, are not able to utilize the DNase-Seq data and are not as suitable as the four methods that we used for benchmarking in this study. However, the methods we benchmarked in this study share the similar concepts with these existing tools (For example, FactorNet is built with similar architecture as the TFImpute with additional support for the DNase-Seq data) and may reflect the potential of them using the same set of features.

The attention weights learned by the models provide an alternative approach to exploring the dependencies between input and output other than saliency maps. By comparing true ChIP-Seq fold change peaks with attention weights, we show how attention weights shift when the fold change peaks move along the DNA sequence. We also demonstrate that our attention model has the ability to learn from known motifs related to specific TFs.

Due to the rules of the DREAM Challenge, we only use very limited types of features in this work. However, if more types of features (such as sequence conservation or epigenetic modifications) are available, they can possibly be transformed into sequential formats and may further improve the prediction performance through our attention architecture. The attention mechanism itself is also evolving rapidly. For example, the multi-head attention introduced by Transformer [37] showed that high-level features could be learned by attention without relying on any recurrent or convolution layers. We expect that better prediction for the TF binding may also be benefited from these novel deep learning architectures in both accuracy and efficacy.

## Conclusions

In this study, we propose a new tool (DeepGRN) that incorporates the attention mechanism with the CNNs-RNNs based architecture. The result shows that the performances of our models are competitive with the top 4 methods in the Challenge leaderboard. We demonstrate that the attention modules in our model help to interpret how critical patterns from different types of input features are recognized.

## Availability and requirements

- Project name: DeepGRN
- Project home page: https://github.com/jianlin-cheng/DeepGRN.
- Operating system(s): Linux, Mac OS, Windows.
- Programming language: Python, R.
- Other requirements: Python version 3.6.0 or higher, R version 3.3.0 or higher.
- License: GNU GPL.
- Any restrictions to use by non-academics: None.

## Supplementary information

**Additional file 1.** Supplementary figures and tables. Including all supplementary figures and tables referenced in the main text.

**Additional file 2.** Instructions of training, prediction, and visualization data with DeepGRN. Including data retrieving, training, prediction with DeepGRN and the implementation details of the visualization scripts used in the main text.

**Additional file 3.** Visualization of the relationship between ChIP-Seq peak and attention weights. For each genomic region, the plot on the left represents the attention weights, and the plot on the right represents the enrichment of ChIP-Seq signal fold changes in the same region. Since the lengths of attention weights are reduced by the convolution and pooling layers, their lengths are less than the fold change values. Thus, the plots are aligned on the X-axis to represent the relative position of fold change and averaged attention weights.

## Data Availability

The datasets used in this study and the source code of DeepGRN are available at https://github.com/jianlin-cheng/DeepGRN.

## Abbreviations

TF: Transcription factor
Bi-LSTM: Bidirectional long short-term memory
DNase-Seq: DNase I hypersensitive sites sequencing
ChIP-Seq: Chromatin immunoprecipitation sequencing
