# Supplementary material for: DeepGRN: prediction of transcription factor binding site across cell-types using attention-based deep neural networks
Source: BMC Bioinformatics. 2021 Feb 1;22:38. doi: 10.1186/s12859-020-03952-1 (PMC7852092; doi:10.1186/s12859-020-03952-1)
Supplement: Supplementary file 1 — Additional file 1. Supplementary figures and tables. Including all supplementary figures and tables referenced in the main text. [file 12859_2020_3952_MOESM1_ESM.pdf]

Figure S1 – The training, hyperparameter tuning, and testing procedure used throughout the Challenge dataset.

The models are trained on the data split described in the methods section. If the leaderboard data is available for a TF, then an additional model selection is performed. The final performance evaluation in this study is performed in the dataset for final ranking determination in the challenge. The models are first trained without epigenomic/conservation scores and external datasets. After training, additional models using epigenomic or conservation score trained using the same configuration as the final model of each TF on all training data. Thus, for each TF there are 6 models in total: pair, single, pair+epigenomic, single+epigenomic, pair+conservation, single+conservation. The results are the combination of two basic models and additional models with auPRC in validation data higher than either of the base models while the downstream analysis are based on two base models.

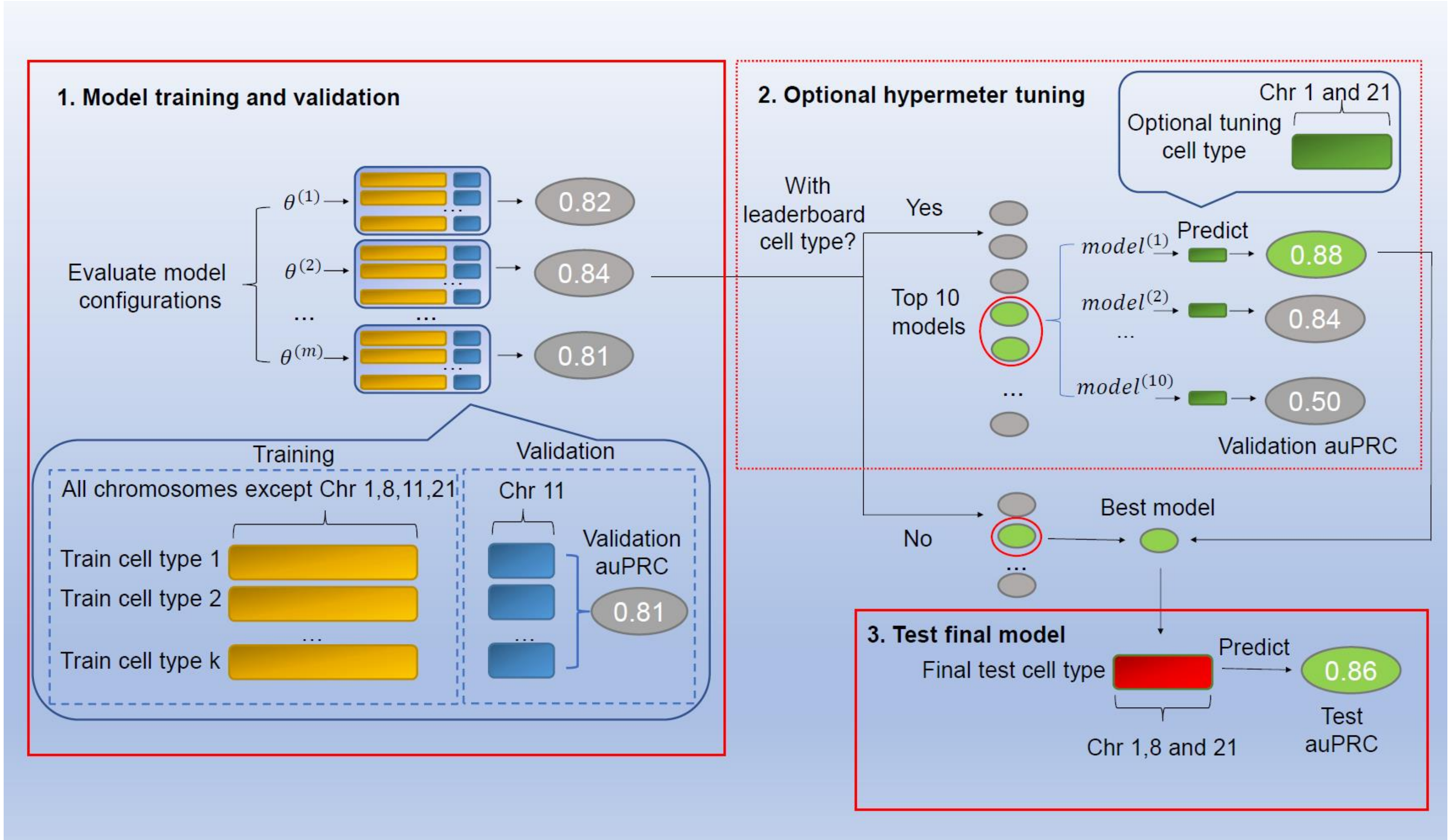

Figure S2 – Performance of DeepGRN with different selections of input ranges.

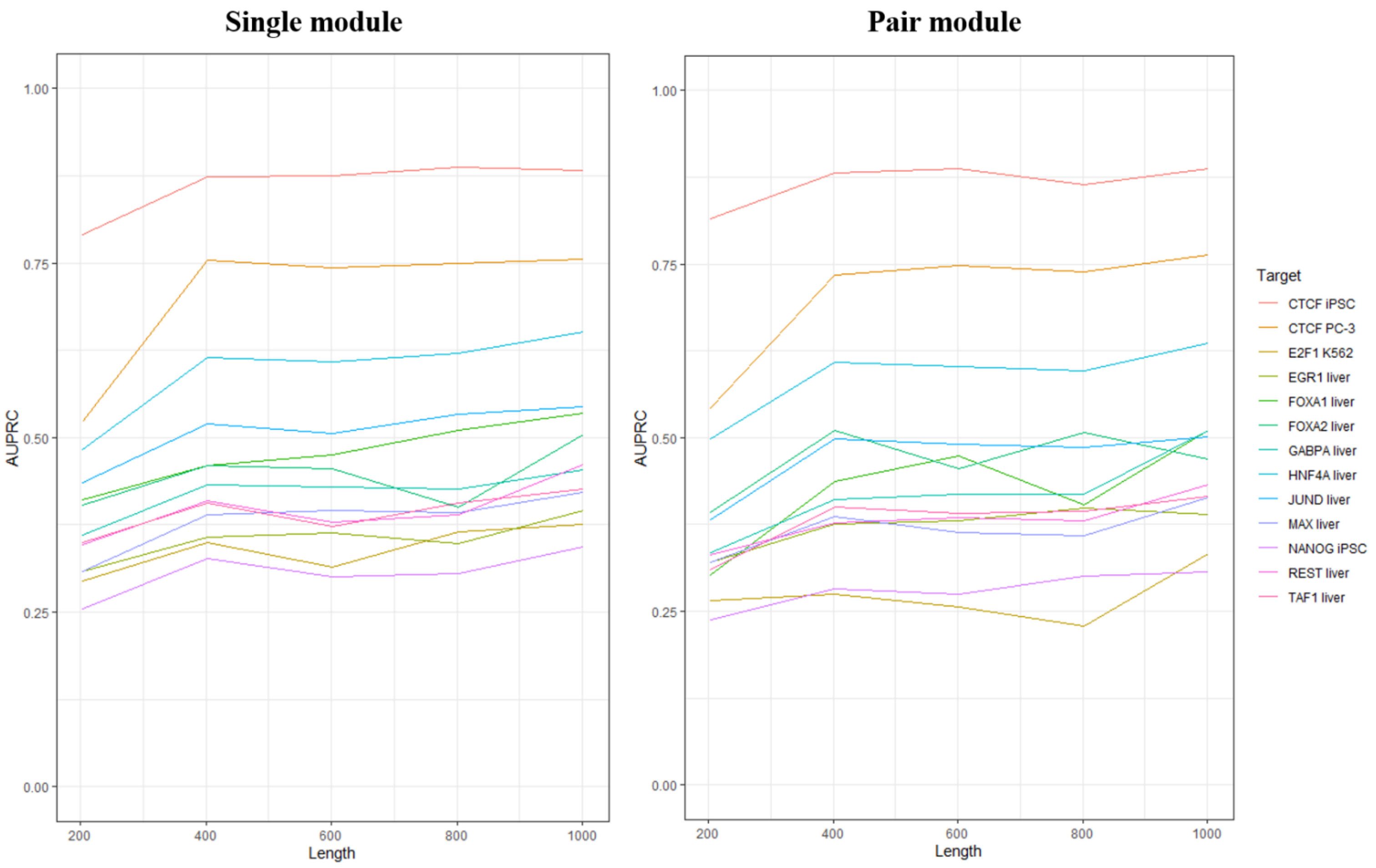

Figure S3 – Comparisons of known motifs and matching motifs learned by the single attention module in CTCF and FOXA1.

(a) Sequence logo built from subsequences detected in CTCF/induced pluripotent cell prediction (left) and motif MA0139.1/ CTCF (right).

(b) The attention scores of the samples selected from CTCF/induced pluripotent cell prediction with hits in FIMO.

(c) The relative positions of the detected motifs in the same region of (b).

(d) The normalized DNase-Seq scores in the same region of (b).

(e) Sequence logo built from subsequences detected in FOXA1/liver cell prediction (left) and motif MA0148.4/ FOXA1 (right).

(f) The attention scores of the samples selected from FOXA1/liver cell prediction with hits in FIMO.

(g) The relative positions of the detected motifs in the same region of (f).

(h) The normalized DNase-Seq scores in the same region of (f).

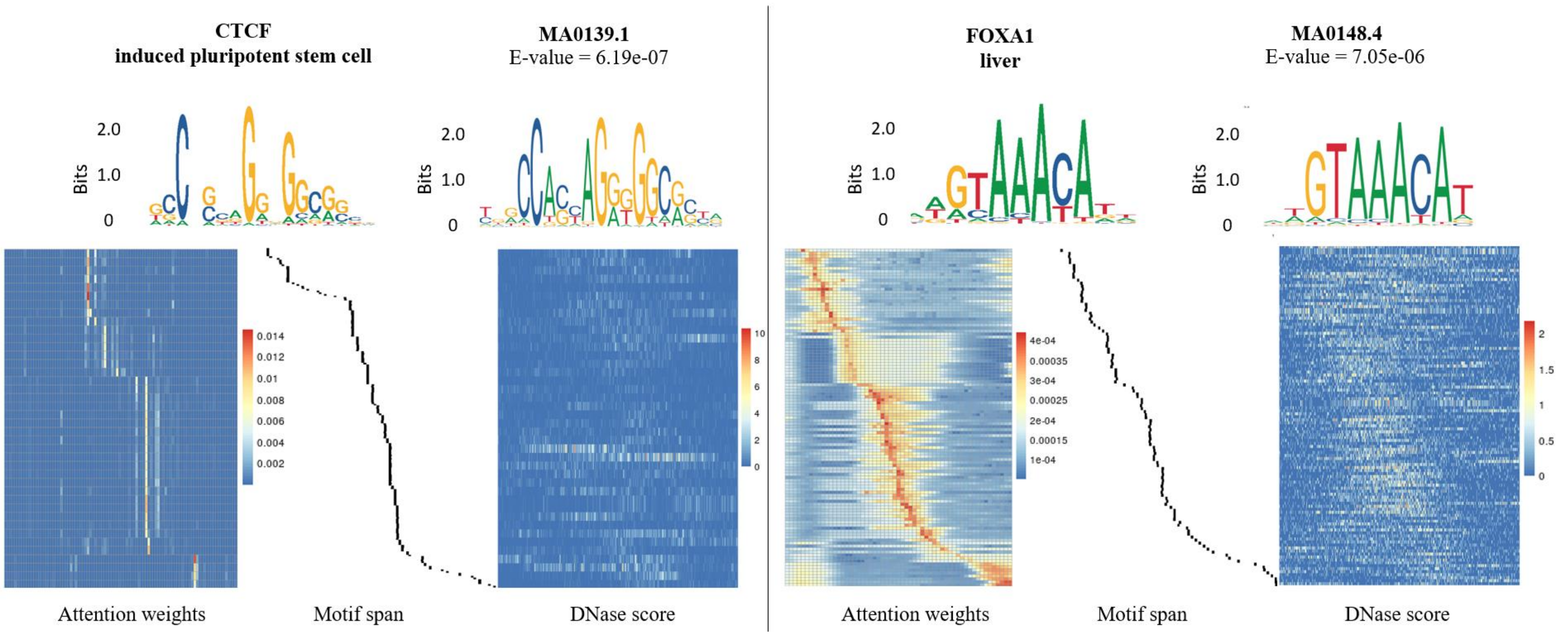

Table S1 – Cell-types selected for training, validation, and evaluation.

TF	Cell Types		
	Training	Optional Model Tuning	Evaluation
CTCF	A549, H1-hESC, HeLa-S3, HepG2, IMR90, K562, MCF-7, GM23338*, HL-60*	GM12878	PC-3, induced pluripotent stem cell
E2F1	GM12878, HeLa-S3		K562
EGR1	GM12878, H1-hESC, HCT116, MCF-7	K562	liver
FOXA1	HepG2	MCF-7	liver
FOXA2	HepG2		liver
GABPA	GM12878, H1-hESC, HeLa-S3, HepG2, MCF-7, HL-60*	K562	liver
HNF4A	HepG2		liver
JUND	HCT116, HeLa-S3, HepG2, K562, MCF-7	H1-hESC	liver
MAX	A549, GM12878, H1-hESC, HCT116, HeLa-S3, HepG2, K562, NB4*	MCF-7	liver
NANOG	H1-hESC, GM23338*		induced pluripotent stem cell
REST	H1-hESC, HeLa-S3, HepG2, MCF-7, Panc1	K562	liver
TAF1	GM12878, H1-hESC, HeLa-S3, K562, GM12891*	HepG2	liver

*: External training datasets derived from the Encode Project and Roadmap Epigenomics.

Table S2 – Hyperparameters selection for model training. Due to the limitation of computing capacity 50 sets of the combination of these hyperparameters are sampled without replacement for evaluating the configurations. The single and pairwise module always share the same configuration.

	Convolution layers				LSTM layers			Attention layer			Dense Layers			
Learning rate	Layers	Kernel Size	Dimension	Dropout rate	Layers	Dimension	Dropout rate	Position	f_{att}	Dimension reduction	Layers	Dimension	Dropout rate	Merge
0.01, 0.001, 0.0005, 0.0001														
	0,1,2													
		15,20, 30,34												
			64,128											
				0.1,0.5										
					1,2									
						32,64								
							0,0.1,0.5							
								Before/ after LSTM						
									MLP, F(x)=x					
										Yes, No				
											2,3,4			
												64,128		
													0.1,0.5	
														Max, Mean

Table S3. The performance of DeepGRN trained with challenge datasets only with four metrics used in the DREAM Challenge.

TF Name	Cell-type	auROC	auPRC	Recall at 50% FDR	Recall at 10% FDR
CTCF	PC-3	0.987	0.764	0.764	0.595
CTCF	induced pluripotent stem cell	0.998	0.891	0.93	0.733
E2F1	K562	0.989	0.376	0.338	0
EGR1	liver	0.993	0.404	0.307	0.019
FOXA1	liver	0.987	0.544	0.579	0.155
FOXA2	liver	0.985	0.539	0.573	0.117
GABPA	liver	0.99	0.506	0.468	0.148
HNF4A	liver	0.978	0.652	0.69	0.303
JUND	liver	0.983	0.542	0.605	0.001
MAX	liver	0.99	0.424	0.34	0.003
NANOG	induced pluripotent stem cell	0.989	0.347	0.314	0.003
REST	liver	0.986	0.47	0.51	0.025
TAF1	liver	0.99	0.425	0.39	0

Table S4. The unified scores of DeepGRN trained with challenge datasets only and the top four algorithms in the DREAM Challenge. Bold scores denote the category that DeepGRN ranks as the highest.

TF	cell	Anchor	FactorNet	Cheburashka	Catchitt	DeepGRN
CTCF	PC-3	0.67	0.17	0.83	0.5	0.33
CTCF	induced pluripotent stem cell	0.83	0.33	0.67	0.5	**0.17**
E2F1	K562	0.33	0.83	0.67	0.17	0.5
EGR1	liver	0.17	0.83	0.67	0.33	0.5
FOXA1	liver	0.67	0.33	0.83	0.5	**0.17**
FOXA2	liver	0.33	0.83	0.67	0.5	**0.17**
GABPA	liver	0.33	0.83	0.67	0.5	**0.17**
HNF4A	liver	0.67	0.33	0.83	0.5	**0.17**
JUND	liver	0.17	0.83	0.67	0.5	0.33
MAX	liver	0.17	0.83	0.33	0.5	0.67
NANOG	induced pluripotent stem cell	0.17	0.5	0.83	0.67	0.33
REST	liver	0.67	0.33	0.83	0.5	**0.17**
TAF1	liver	0.17	0.5	0.83	0.33	0.67

Table S5 – Individual performance of single and pairwise attention module. For auROC and auPRC, bold values denote a higher score in the corresponding module. Both results are from base models trained with challenge datasets only.

TF Name	Cell type	Single attention				Pairwise attention			
		auROC	auPRC	Re@0.50 FDR	Re@0.10 FDR	auROC	auPRC	Re@0.50 FDR	Re@0.10 FDR
CTCF	PC-3	0.985	0.756	0.762	0.583	**0.989**	0.**763**	0.76	0.59
CTCF	induced pluripotent stem cell	0.998	0.883	0.923	0.712	0.998	**0.888**	0.925	0.73
E2F1	K562	0.99	**0.375**	0.378	0	0.983	0.333	0.27	0
EGR1	liver	0.992	**0.396**	0.309	0.026	0.992	0.39	0.281	0.002
FOXA1	liver	**0.989**	**0.535**	0.565	0.115	0.98	0.511	0.53	0.152
FOXA2	liver	**0.986**	**0.504**	0.527	0.052	0.981	0.469	0.481	0.055
GABPA	liver	0.987	0.454	0.382	0.134	**0.99**	**0.511**	0.479	0.135
HNF4A	liver	**0.98**	**0.652**	0.699	0.286	0.973	0.636	0.679	0.273
JUND	liver	0.981	**0.545**	0.615	0.013	**0.982**	0.502	0.557	0
MAX	liver	**0.991**	**0.422**	0.322	0.001	0.988	0.414	0.326	0.003
NANOG	induced pluripotent stem cell	**0.99**	**0.343**	0.292	0	0.98	0.307	0.261	0.001
REST	liver	**0.986**	**0.461**	0.469	0.009	0.984	0.433	0.376	0.028
TAF1	liver	0.989	**0.426**	0.391	0	0.989	0.416	0.361	0.002
