# Supplementary material for: DeepGRN: prediction of transcription factor binding site across cell-types using attention-based deep neural networks
Source: BMC Bioinformatics. 2021 Feb 1;22:38. doi: 10.1186/s12859-020-03952-1 (PMC7852092; doi:10.1186/s12859-020-03952-1)
Supplement: Supplementary file 2 — Additional file 2. Instructions of training, prediction, and visualization data with DeepGRN. Including data retrieving, training, prediction with DeepGRN and the implementation details of the visualization scripts used in the main text. [file 12859_2020_3952_MOESM2_ESM.pdf]

Instructions of training, prediction, and visualization data with DeepGRN

Chen Chen 3/1/2020

This file includes retrieving data used in training, prediction with DeepGRN and the implementation details of the visualization scripts used in the main text. All codes mentioned in this section can be found at https://github.com/jianlin-cheng/DeepGRN.

Download and process the input data for DeepGRN (using ENCODE-DREAM data as example):

Assuming all feature data will be generated at $DATAPATH (e.g. /home/yourname/data/inputs/) Create directory structures

```
mkdir -p $DATAPATHbams
mkdir -p $DATAPATHDNase
mkdir -p $DATAPATHchipseq
mkdir -p $DATAPATHgencode
mkdir -p $DATAPATHlabel/train
mkdir -p $DATAPATHlabel/leaderboard
mkdir -p $DATAPATHlabel/final
mkdir -p $DATAPATHlabel/train_positive
mkdir -p $DATAPATHlabel/train_negative
```

Download annotation files for genomic elements

```
cd $DATAPATHgencode
wget https://github.com/uci-
cbcl/FactorNet/raw/master/resources/wgEncodeGencodeBasicV19.cds.merged.bed.g
wget https://github.com/uci-
cbcl/FactorNet/raw/master/resources/wgEncodeGencodeBasicV19.intron.merged.be
wget https://github.com/uci-
cbcl/FactorNet/raw/master/resources/wgEncodeGencodeBasicV19.promoter.merged.
wget https://github.com/uci-
cbcl/FactorNet/raw/master/resources/wgEncodeGencodeBasicV19.txStart.bed.gz
wget https://github.com/uci-
cbcl/FactorNet/raw/master/resources/wgEncodeGencodeBasicV19.utr3.merged.bed.
wget https://github.com/uci-
cbcl/FactorNet/raw/master/resources/wgEncodeGencodeBasicV19.utr5.merged.bed.
```

Get data from synapse(ChIP-Seq files, DNase files)

```
python get_syn_data.py $DATAPATH
```

Generate region file for prediction

```
cd $DATAPATHlabel/
gzip -d *.gz
awk ’$1 == "chr1" {print}’ test_regions.blacklistfiltered.bed > predict_region.bed
awk ’$1 == "chr21" {print}’ test_regions.blacklistfiltered.bed >> predict_region.bed
```

```
awk '$1 == "chr8" {print}' test_regions.blacklistfiltered.bed >> predict_region.bed
```

Download blacklist file, genome file, and uniqueness file from FactorNet repository since we also need to process the data by the way Factornet did.

```
cd $DATAPATH
wget https://github.com/uci-cbcl/FactorNet/raw/master/resources/blacklist.bed.gz
wget https://raw.githubusercontent.com/uci-cbcl/FactorNet/master/resources/hg19.autoX.chrom.sizes
# get unique 35 data
wget
http://hgdownload.cse.ucsc.edu/goldenpath/hg19/encodeDCC/wgEncodeMapability/wgEncodeDukeMapab
ilityU
wget
http://hgdownload.cse.ucsc.edu/goldenpath/hg19/encodeDCC/wgEncodeMapability/wgEncodeDacMapabili
tyCo
```

Generate 2 missing bigwig files(A549 and IMR-90) from bams

```
cd $DATAPATHbams
samtools merge A549.bam DNASE.A549.*
samtools index A549.bam
bamCoverage --bam A549.bam -o ../DNase/A549.1x.bw --outFileFormat bigwig --normalizeTo1x
2478297382 --ig
samtools merge IMR-90.bam DNASE.IMR90.*
samtools index IMR-90.bam
bamCoverage --bam IMR-90.bam -o ../DNase/IMR-90.1x.bw --outFileFormat bigwig --normalizeTo1x
2478297382
```

Generate masks for genomic elements and blacklists, these can be user defined as long as they are binary(0,1) vectors with the same length with the training/prediction bins. For genomic elements, 0 means not overlapping and 1 means overlapping for each bin. For blacklists zero means overlapping with black list. Those bins will not be trained and force to be 0 in prediction.

```
cd $DATAPATH../src
python get_mask_files.py
```

get_rawdata/get_data.sh provided a demo to generate all the required data for the Dream-Encode Challenge 2016 in one step:

```
bash get_rawdata/get_data.sh /home/yourname/data/inputs/
```

Required softwares:

To use DeepGRN on your local machine, please make sure the following required softwares are already installed:

Python 3.6.6
bedtools 2.26.0

Python modules:

pandas 0.24.2

numpy 1.16.2
tensorflow 1.11.0
pyfaidx 0.5.5.2
pyfasta 0.5.2
pybedtools 0.8.0
pyBigWig 0.3.14
Keras 2.2.4
h5py 2.9.0
deepTools 3.2.1

Optional(for training with GPUs):

cuda 10
tensorflow-gpu 1.11.0

Training

(train.py)
Train models for TF binding site prediction:

usage: train.py [-h] --data_dir DATA_DIR --tf_name TF_NAME --output_dir
    OUTPUT_DIR [--attention_position ATTENTION_POSITION]

Arguments

[--flanking FLANKING] [--val_chr VAL_CHR] [--epochs EPOCHS]
[--patience PATIENCE] [--batch_size BATCH_SIZE]
[--learningrate LEARNINGRATE] [--kernel_size KERNEL_SIZE]
[--num_filters NUM_FILTERS] [--num_recurrent NUM_RECURRENT]
[--num_dense NUM_DENSE] [--dropout_rate DROPOUT_RATE]
[--rnn_dropout1 RNN_DROPOUT1] [--rnn_dropout2 RNN_DROPOUT2]
[--merge MERGE] [--num_conv NUM_CONV] [--num_lstm NUM_LSTM]
[--num_denselayer NUM_DENSELAYER]
[--ratio_negative RATIO_NEGATIVE] [--rnaseq] [--gencode]
[--unique35] [--conservative] [--cgi_score] [--use_peak] [--use_cudnn]
[--single_attention_vector] [--plot_model]
[--random_seed RANDOM_SEED]

- -h, --help show this help message and exit
- --data_dir DATA_DIR, -i DATA_DIR data path
- --tf_name TF_NAME, -t TF_NAME name of TF
- --output_dir OUTPUT_DIR, -o OUTPUT_DIR output dir
- --attention_position ATTENTION_POSITION, -ap ATTENTION_POSITION Position of attention lay-

  ers, can be attention1d_after_lstm, attention_before_lstm,attention_after_lstm,attention1d_after_lstm

- --flanking FLANKING, -f FLANKING flanking
- --val_chr VAL_CHR, -v VAL_CHR validation chr
- --epochs EPOCHS, -e EPOCHS epochs
- --patience PATIENCE, -p PATIENCE patience for training cycle
- --batch_size BATCH_SIZE, -s BATCH_SIZE batch size
- --learningrate LEARNINGRATE, -l LEARNINGRATE learning rate

- --kernel_size KERNEL_SIZE, -k KERNEL_SIZE kernel_size for Conv1D

- --num_filters NUM_FILTERS, -nf NUM_FILTERS number of filters for Conv1D
- --num_recurrent NUM_RECURRENT, -nr NUM_RECURRENT Output dim for LSTM
- --num_dense NUM_DENSE, -nd NUM_DENSE Output dim for dense layers
- --dropout_rate DROPOUT_RATE, -d DROPOUT_RATE dropout rate for all layers except LSTM
- --rnn_dropout1 RNN_DROPOUT1, -rd1 RNN_DROPOUT1 dropout rate for LSTM
- --rnn_dropout2 RNN_DROPOUT2, -rd2 RNN_DROPOUT2 rnn dropout rate for LSTM
- --merge MERGE, -me MERGE merge method, max or ave
- --num_conv NUM_CONV, -nc NUM_CONV Number of Conv1D layers
- --num_lstm NUM_LSTM, -nl NUM_LSTM Number of LSTM layers
- --num_denselayer NUM_DENSELAYER, -dl NUM_DENSELAYER Number of additional dense layers
- --ratio_negative RATIO_NEGATIVE, -rn RATIO_NEGATIVE Ratio of negative samples to positive

  samples in each epoch

- --rnaseq, -r should rnaseq data be used
- --gencode, -g should gencode data be used
- --unique35, -u should unique35 data be used
- --conservative, -cv should conservation data be used
- --cgi_score, -ci should CGI score be used
- --use_peak, -a should the training be performed in “augmented” style
- --use_cudnn, -c use cudnnLSTM instead of LSTM, faster but will disable LSTM dropouts
- --single_attention_vector, -sa merge attention weights in each position by averaging
- --plot_model, -pl plot model as png file
- --random_seed RANDOM_SEED, -rs RANDOM_SEED random seed

Prediction

(predict.py)
Use models trained from train.py for new data prediction:

usage: predict.py [-h] --data_dir DATA_DIR --model_file MODEL_FILE --cell_name
        CELL_NAME --predict_region_file PREDICT_REGION_FILE

Predict a model.

Arguments

--output_predict_path OUTPUT_PREDICT_PATH
[--batch_size BATCH_SIZE] [--blacklist_file BLACKLIST_FILE]

- -h, --help show this help message and exit
- --data_dir DATA_DIR, -i DATA_DIR data path
- --model_file MODEL_FILE, -m MODEL_FILE model file
- --cell_name CELL_NAME, -c CELL_NAME cell name
- --predict_region_file PREDICT_REGION_FILE, -p PREDICT_REGION_FILE predict region file
- --output_predict_path OUTPUT_PREDICT_PATH, -o OUTPUT_PREDICT_PATH output for predict
- --batch_size BATCH_SIZE, -b BATCH_SIZE batch size
- --blacklist_file BLACKLIST_FILE, -l BLACKLIST_FILE blacklist file to use, can be left empty
