# Supplementary material for: DeepGRN: prediction of transcription factor binding site across cell-types using attention-based deep neural networks
Source: BMC Bioinformatics. 2021 Feb 1;22:38. doi: 10.1186/s12859-020-03952-1 (PMC7852092; doi:10.1186/s12859-020-03952-1)
Supplement: Supplementary file 3 — Additional file 3. Visualization of the relationship between ChIP-Seq peak and attention weights. For each genomic region, the plot on the left represents the attention weights, and the plot on the right represents the enrichment of ChIP-Seq signal fold changes in the same region. Since the lengths of attention weights are reduced by the convolution and pooling layers, their lengths are less than the fold change values. Thus, the plots are aligned on the X-axis to represent the relative position of fold change and averaged attention weights. [file 12859_2020_3952_MOESM3_ESM.pdf]

Visualization of the relationship between ChIP-seq peak and attention weights.

For each genomic region, the figure on the left represents the attention weights and the figure on the right represents the enrichment of fold changes in ChIP-seq BigWig file in the same region. Since the lengths of attention weights are reduced by the convolution and pooling layers, their lengths are less than the fold change values. Thus, the plots are aligned on the X-axis to represent the relative position of fold change and averaged attention weights.

The attention scores are extracted from both single and pairwise models. For single module, the dimension of attention scores is $L \times d_{model}$, and the channel that contains the highest attention weight is used. Similarly, for pairwise module, the dimension of attention scores is $L \times L$, and the row sum of these weights are always 1. As a result, we use the row that contains the highest attention weight.

Single attention module

CTCF/induced pluripotent stem cell

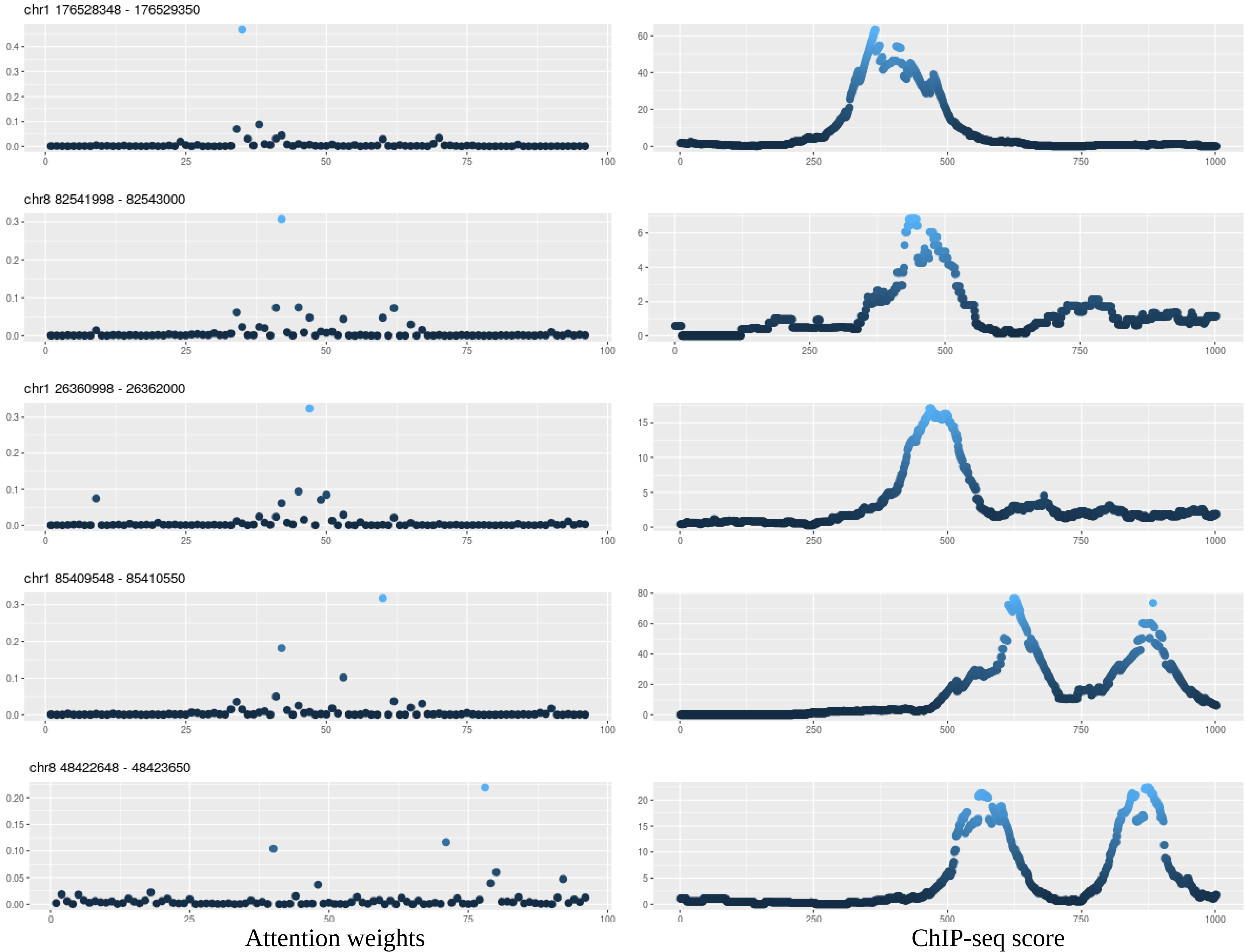

Single attention module

CTCF/PC-3

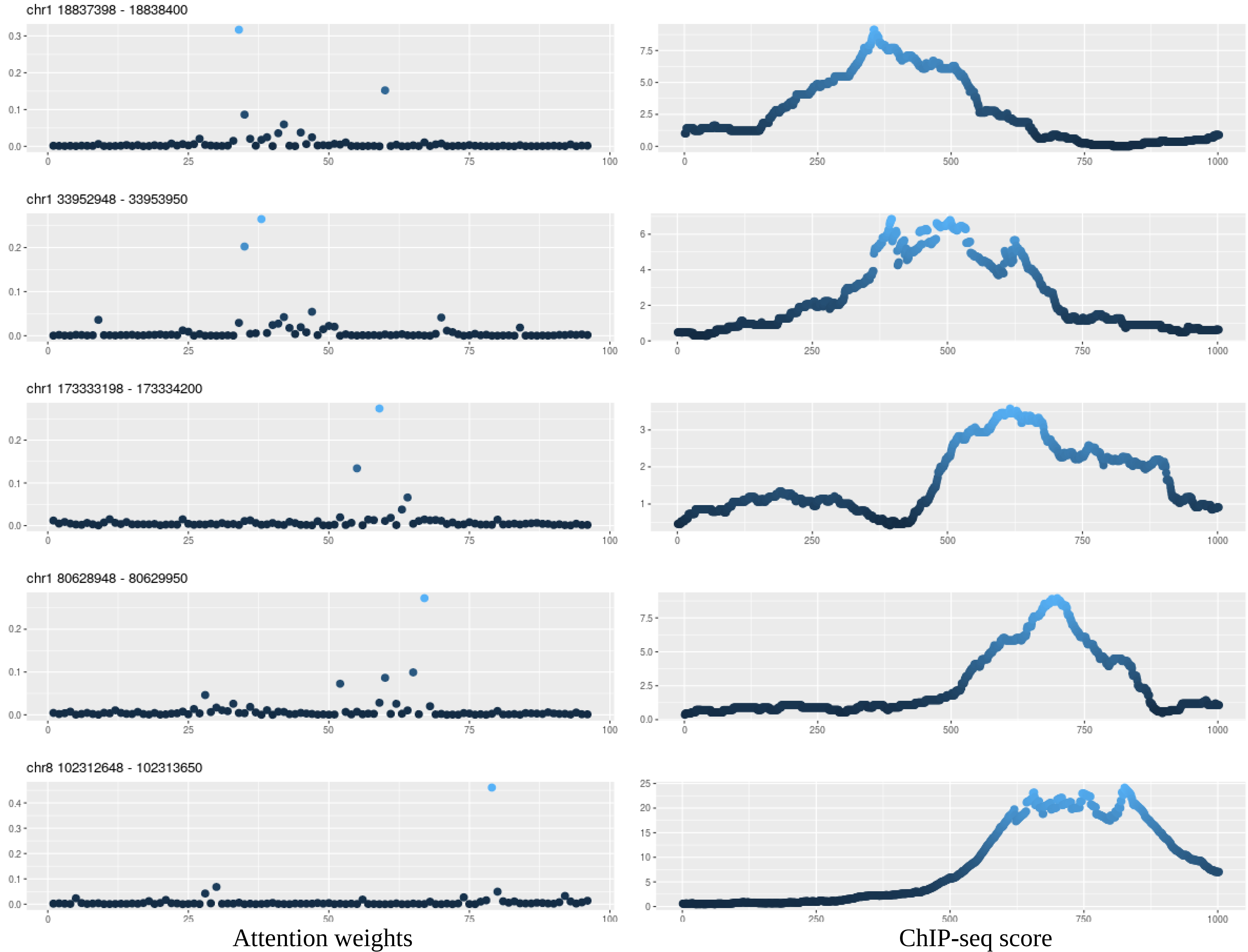

Single attention module

E2F1/K562

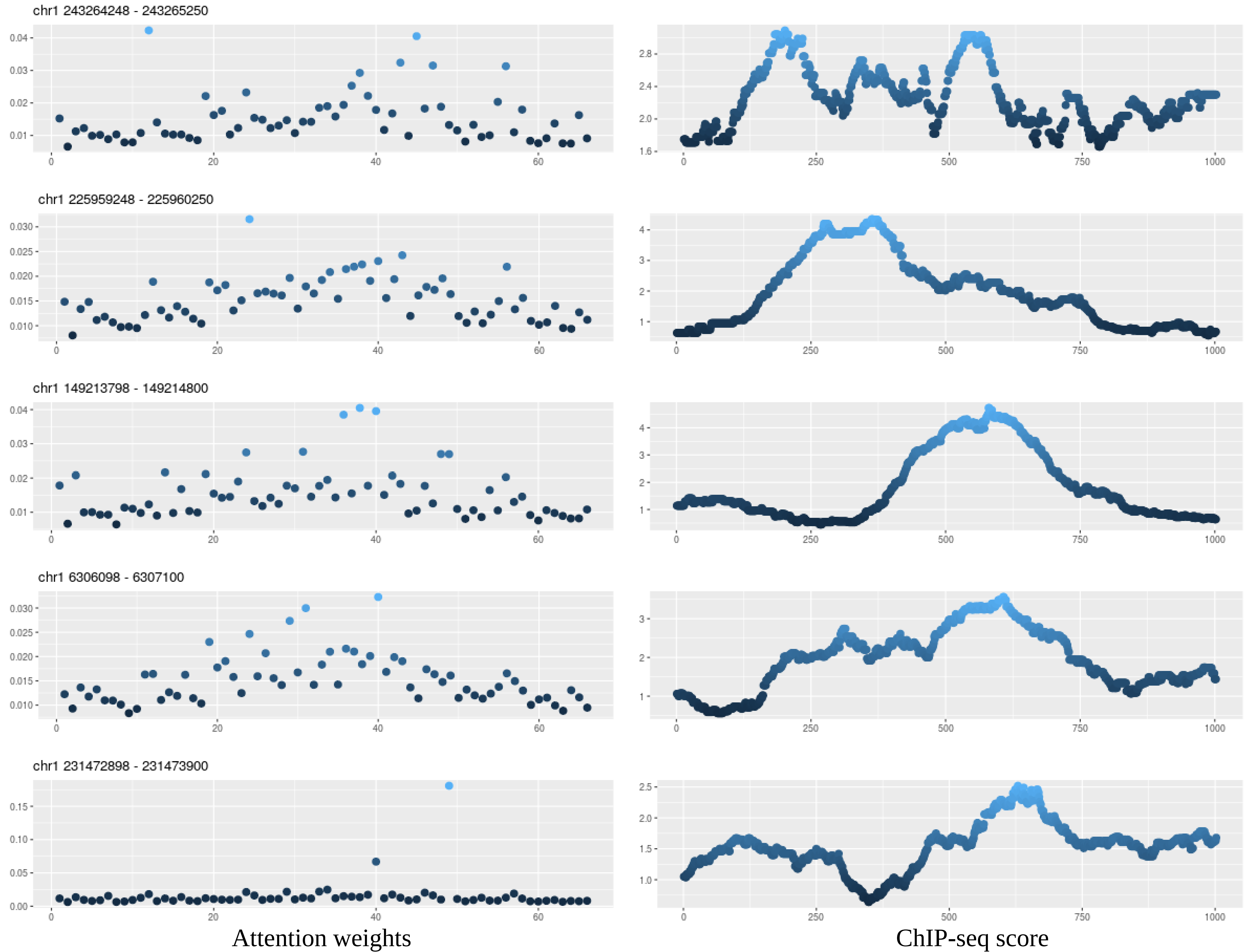

Single attention module

EGR1/liver

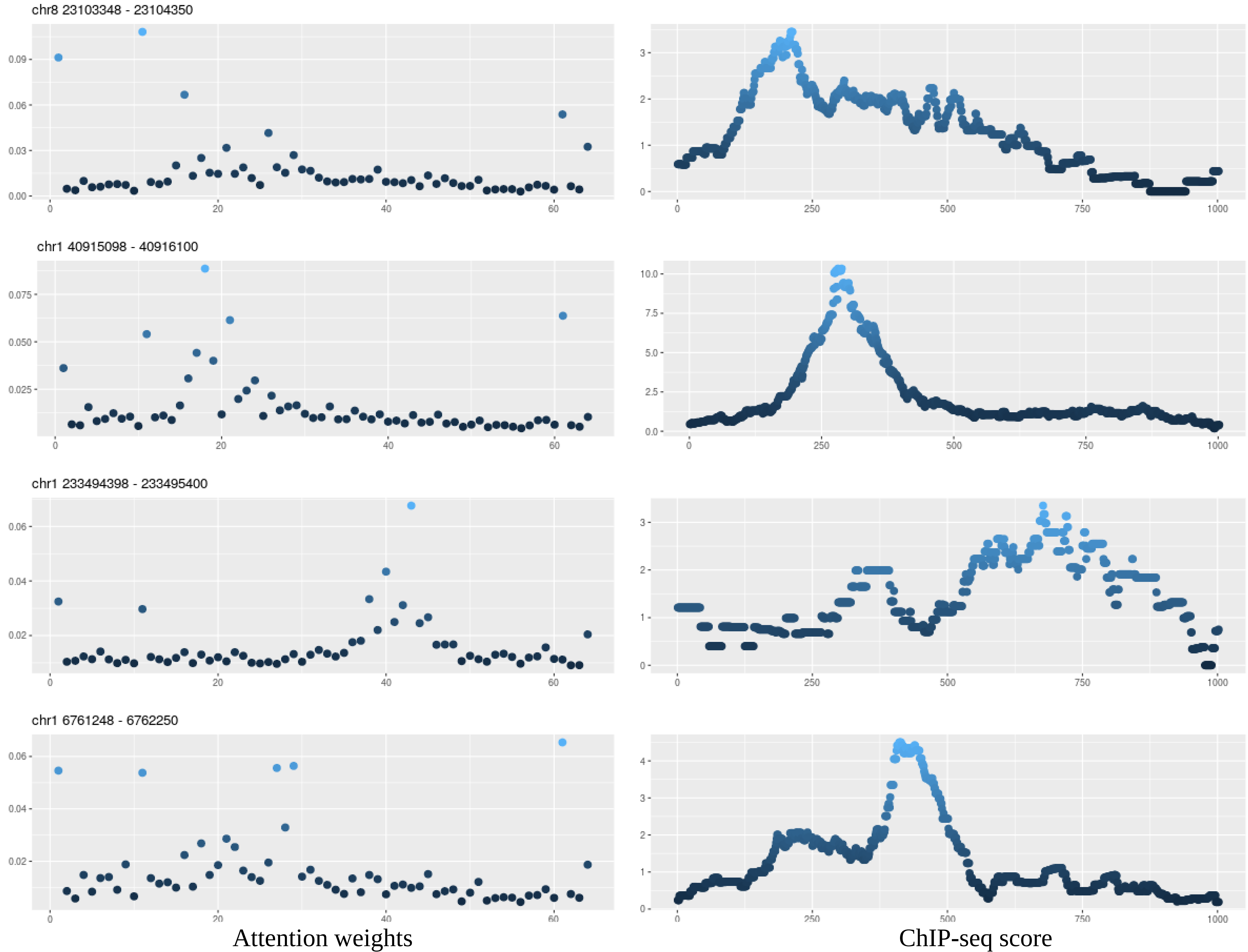

Single attention module

FOXA1/liver

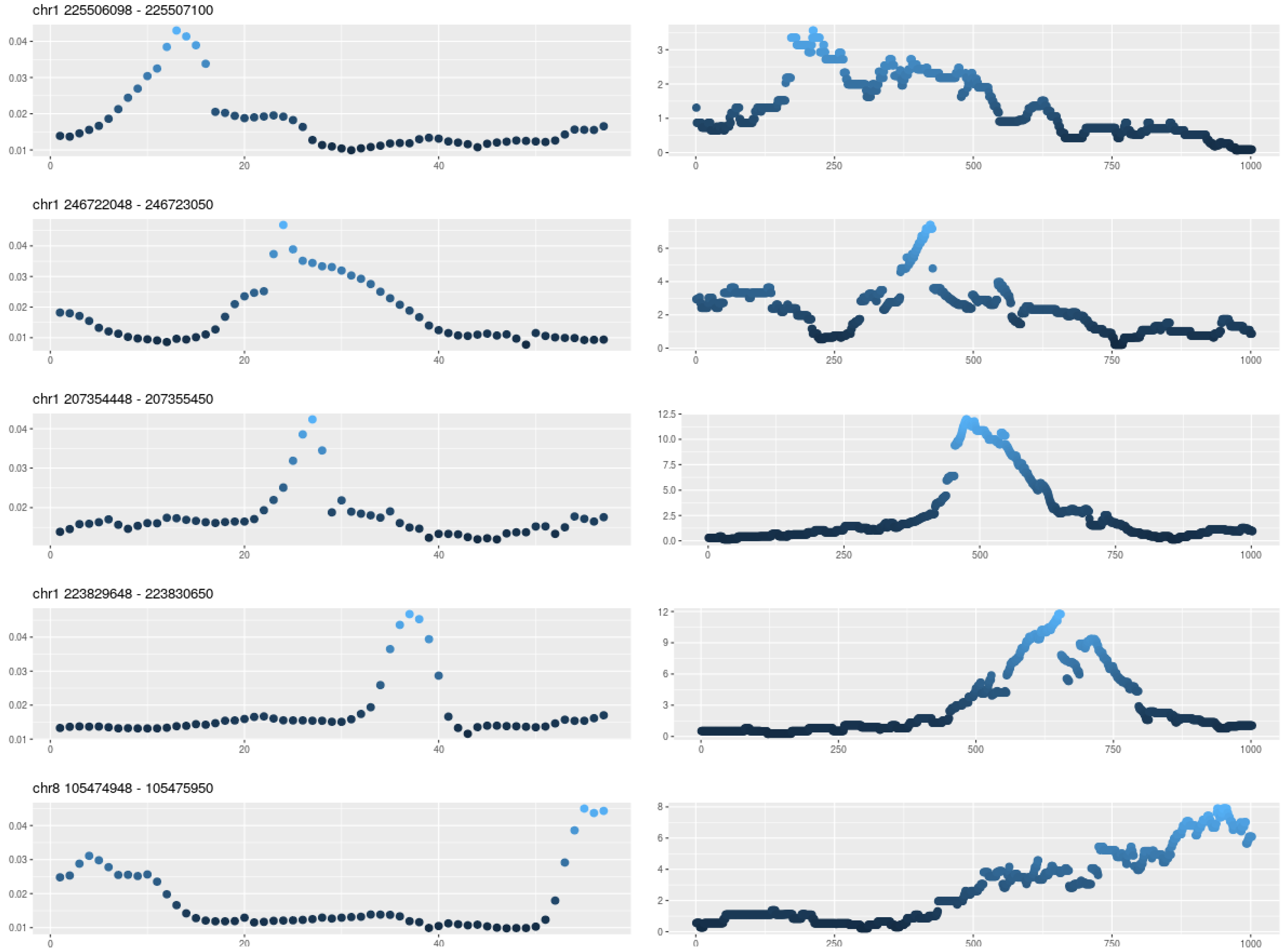

Attention weights ChIP-seq score

Single attention module

FOXA2/liver

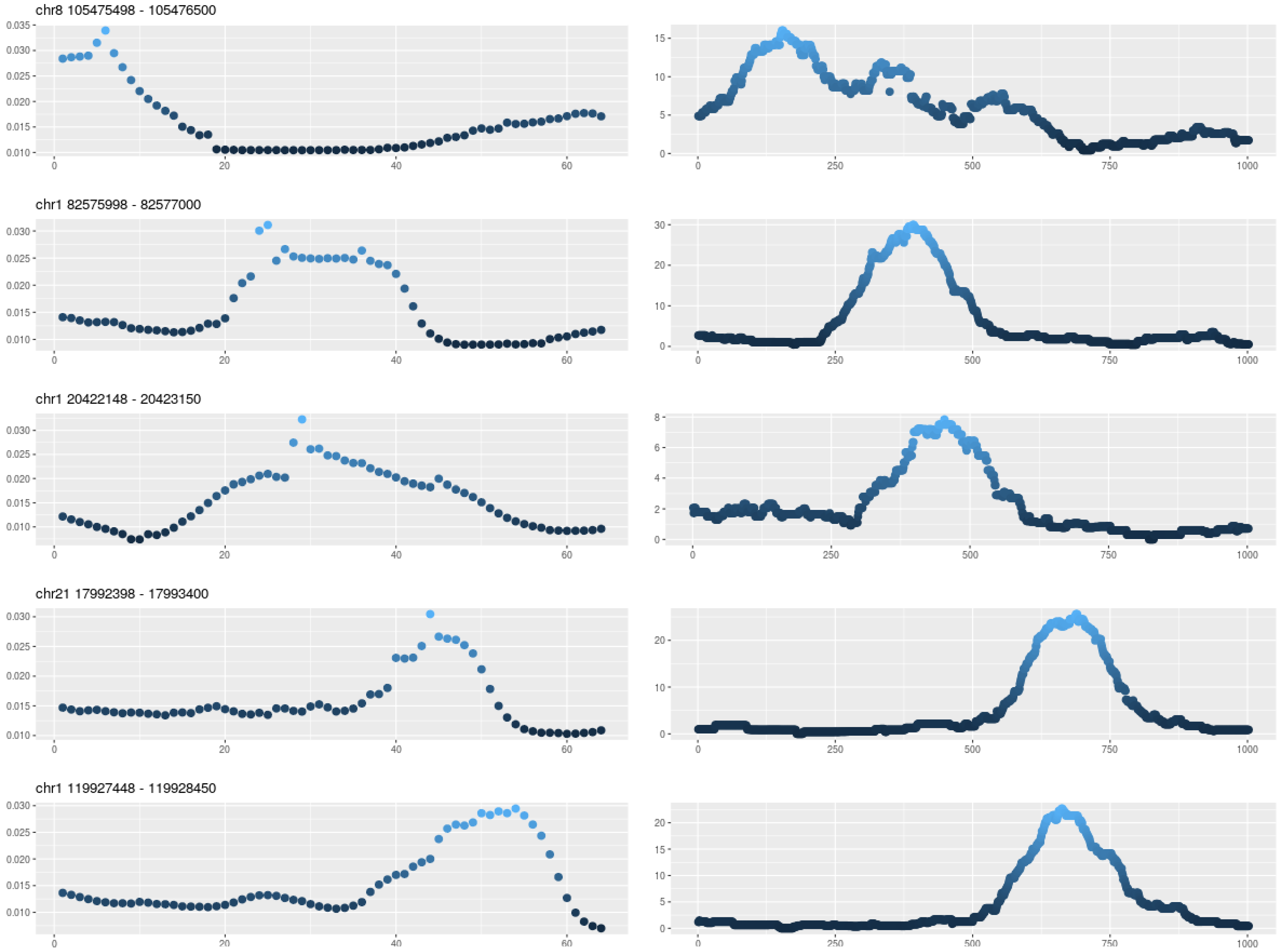

Attention weights ChIP-seq score

Single attention module

GABPA/liver

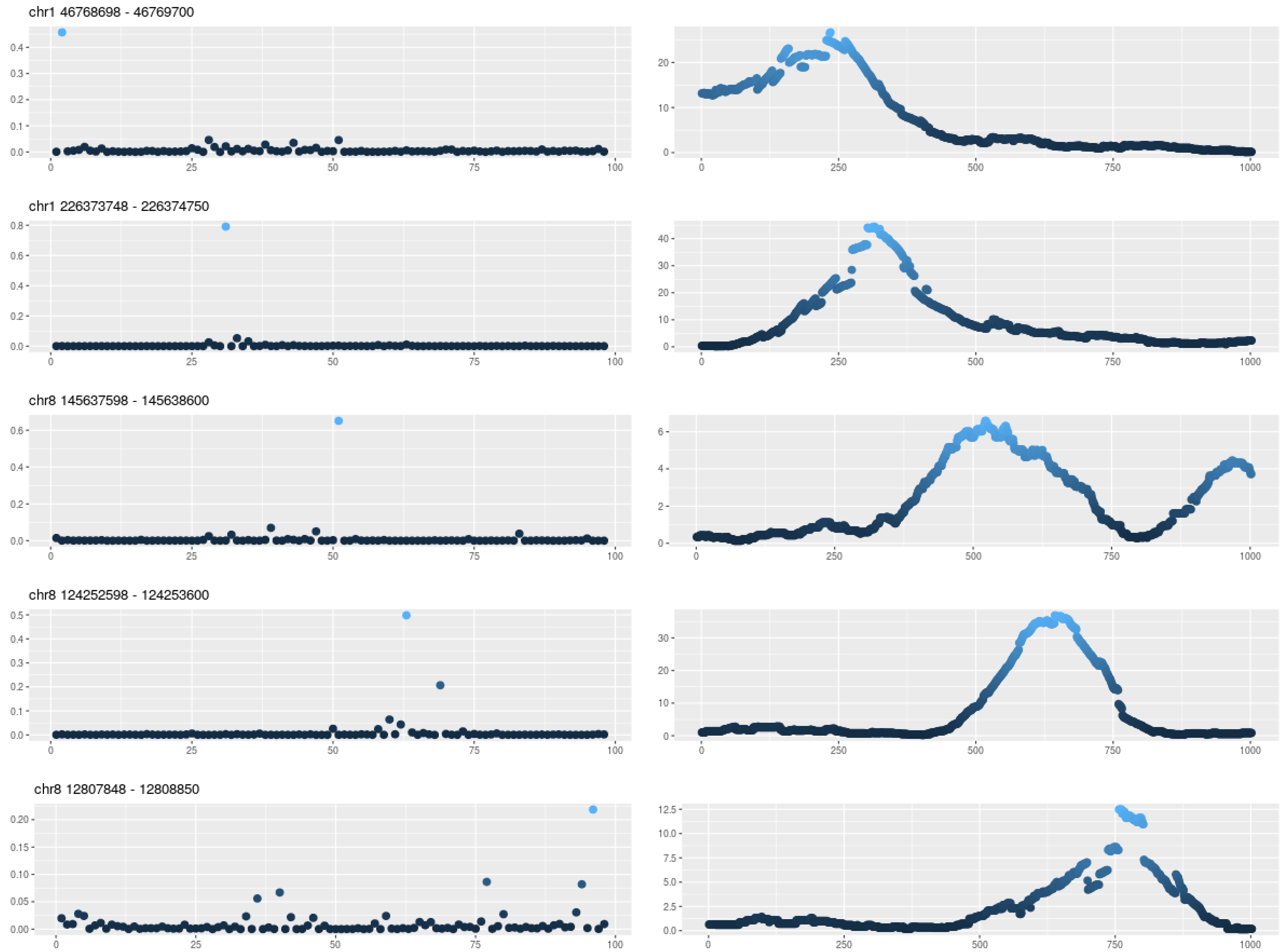

Attention weights ChIP-seq score

Single attention module

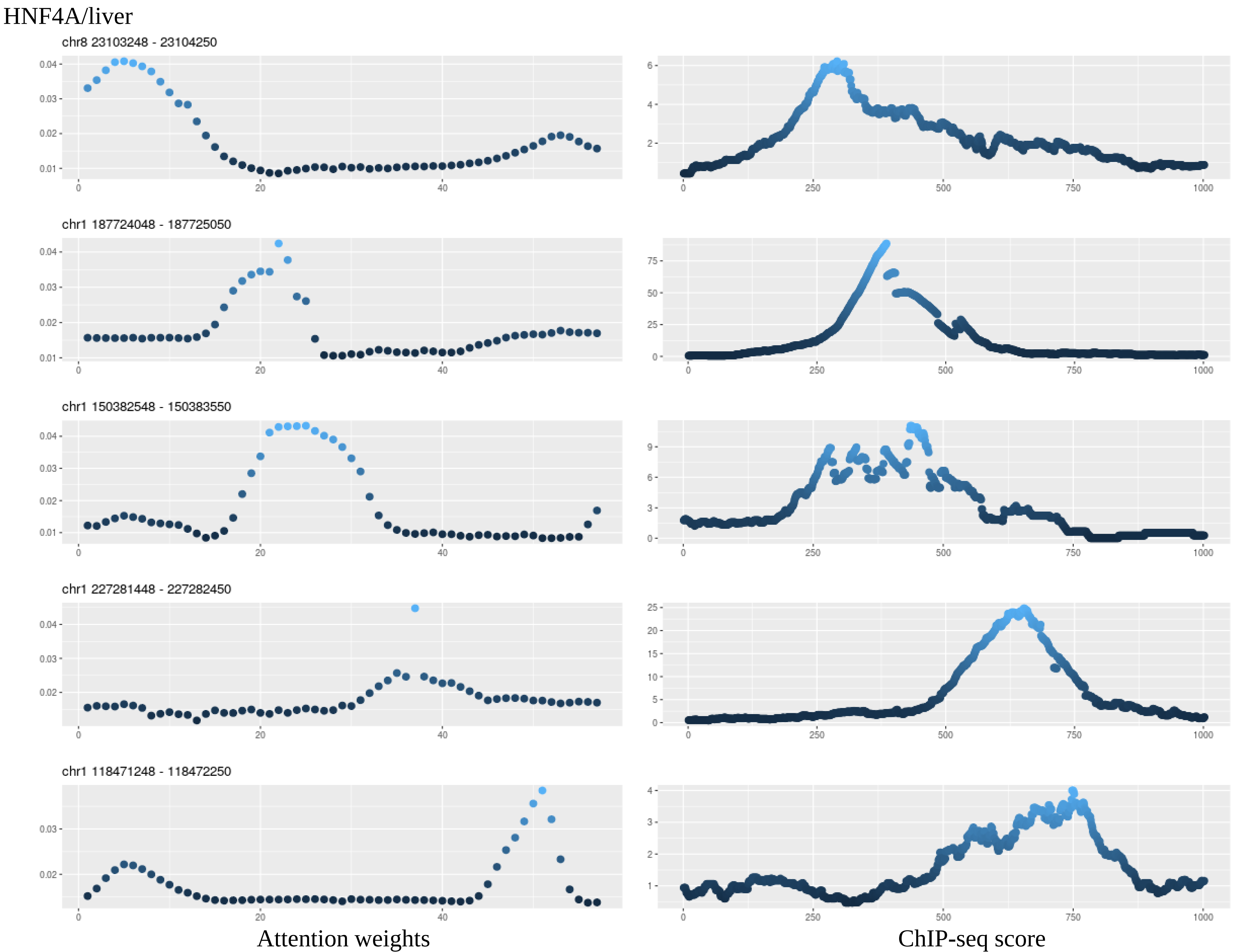

Single attention module

JUND/liver

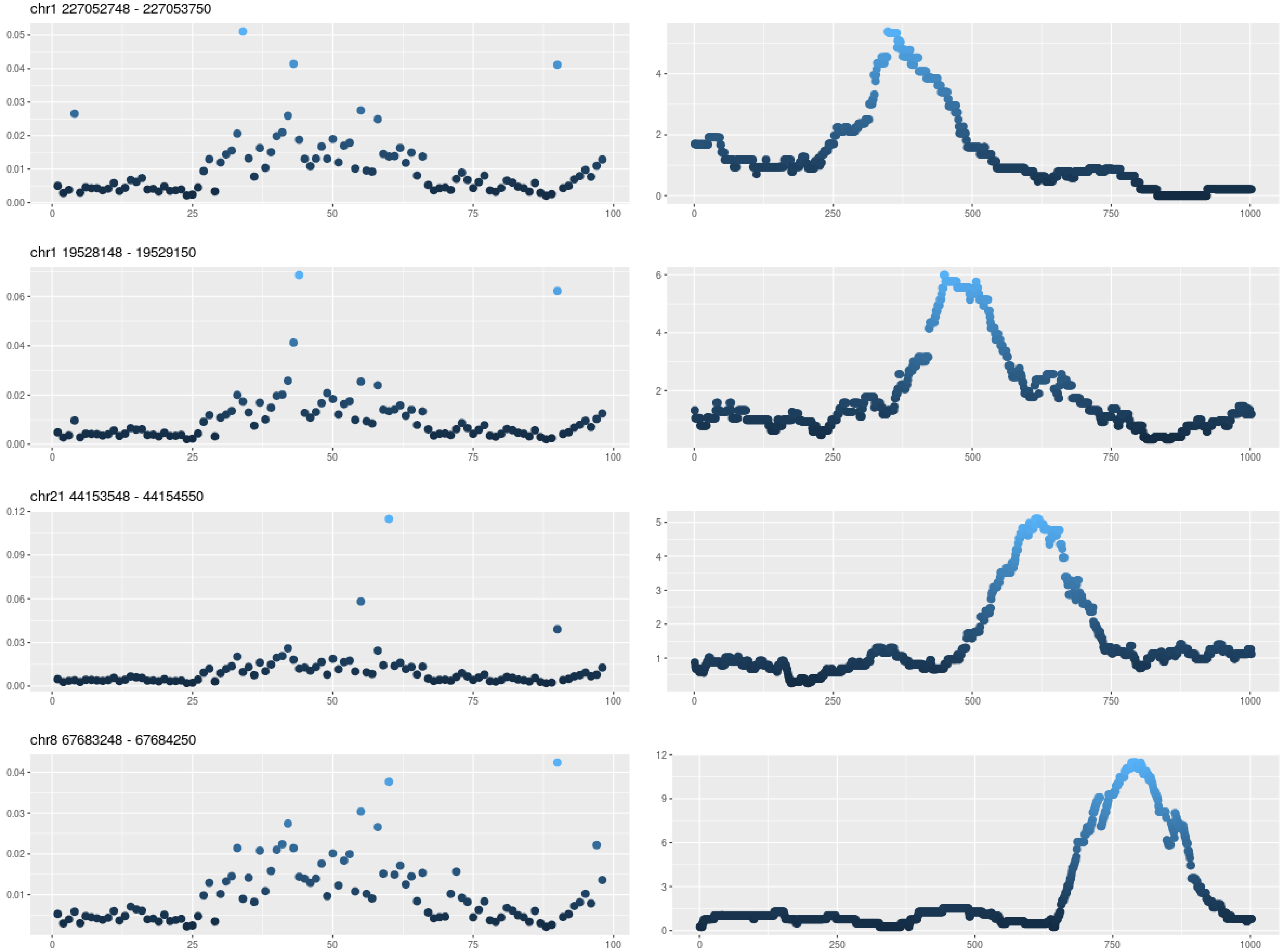

Attention weights ChIP-seq score

Single attention module

MAX/liver

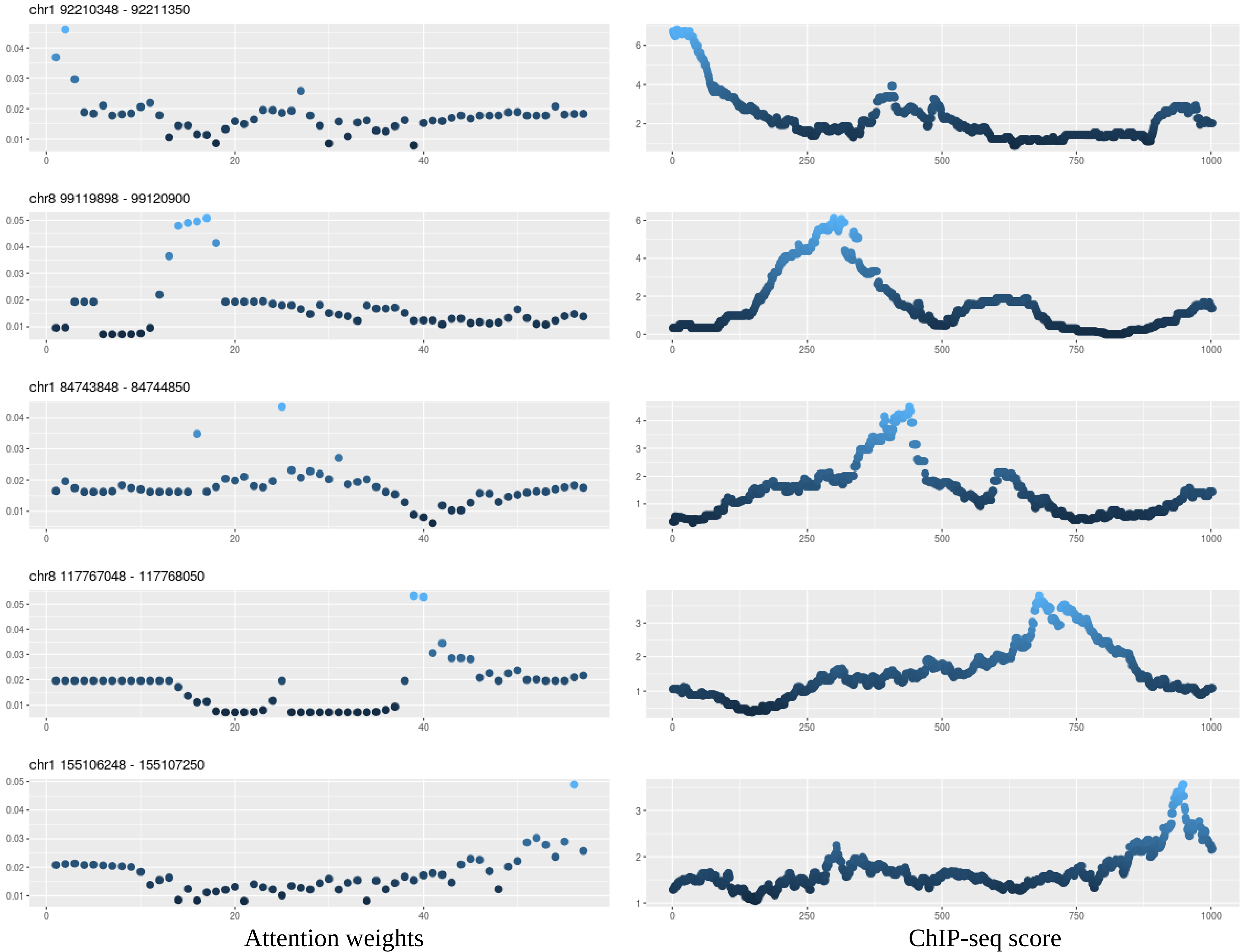

Single attention module

NANOG/induced pluripotent stem cell

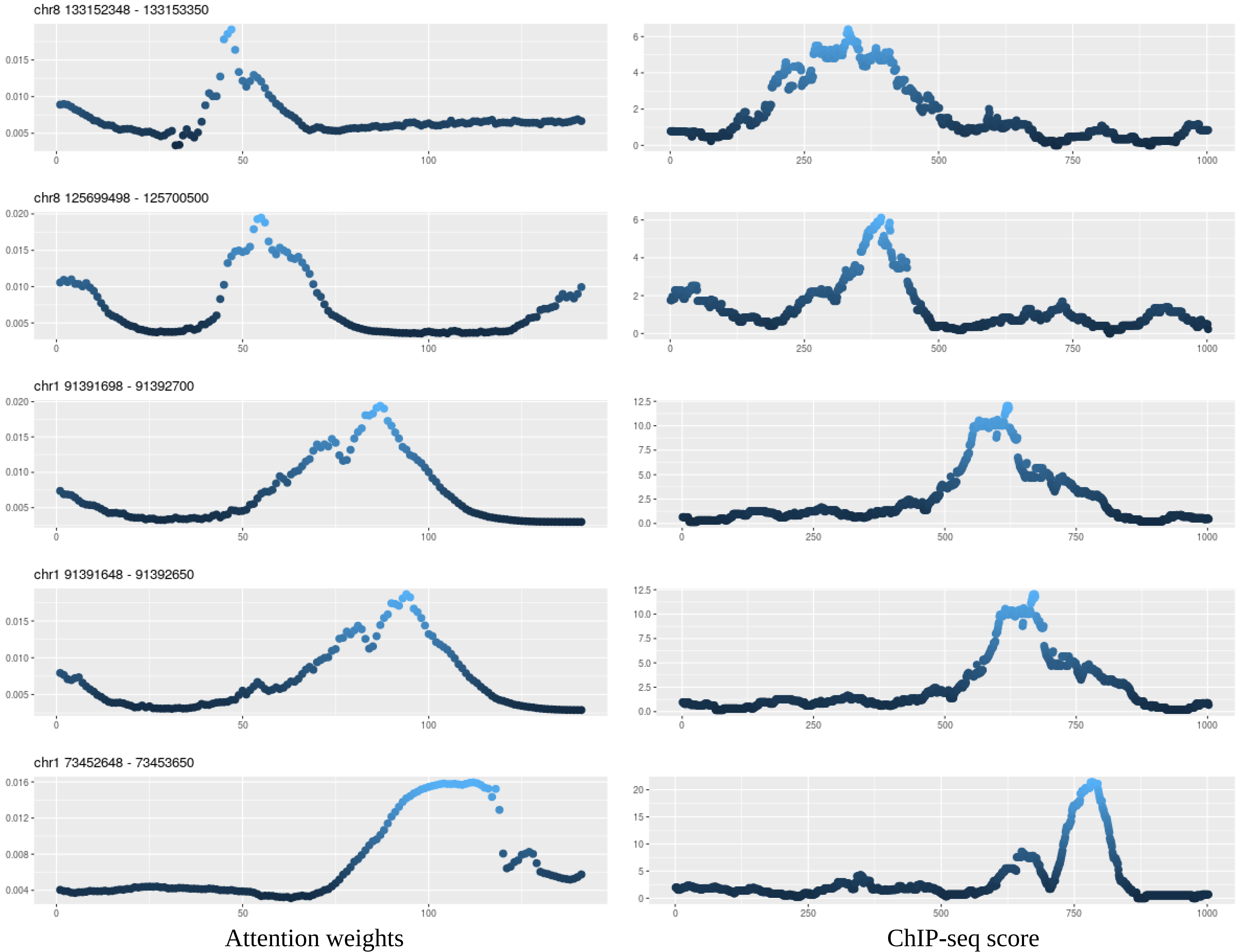

Single attention module

REST/liver

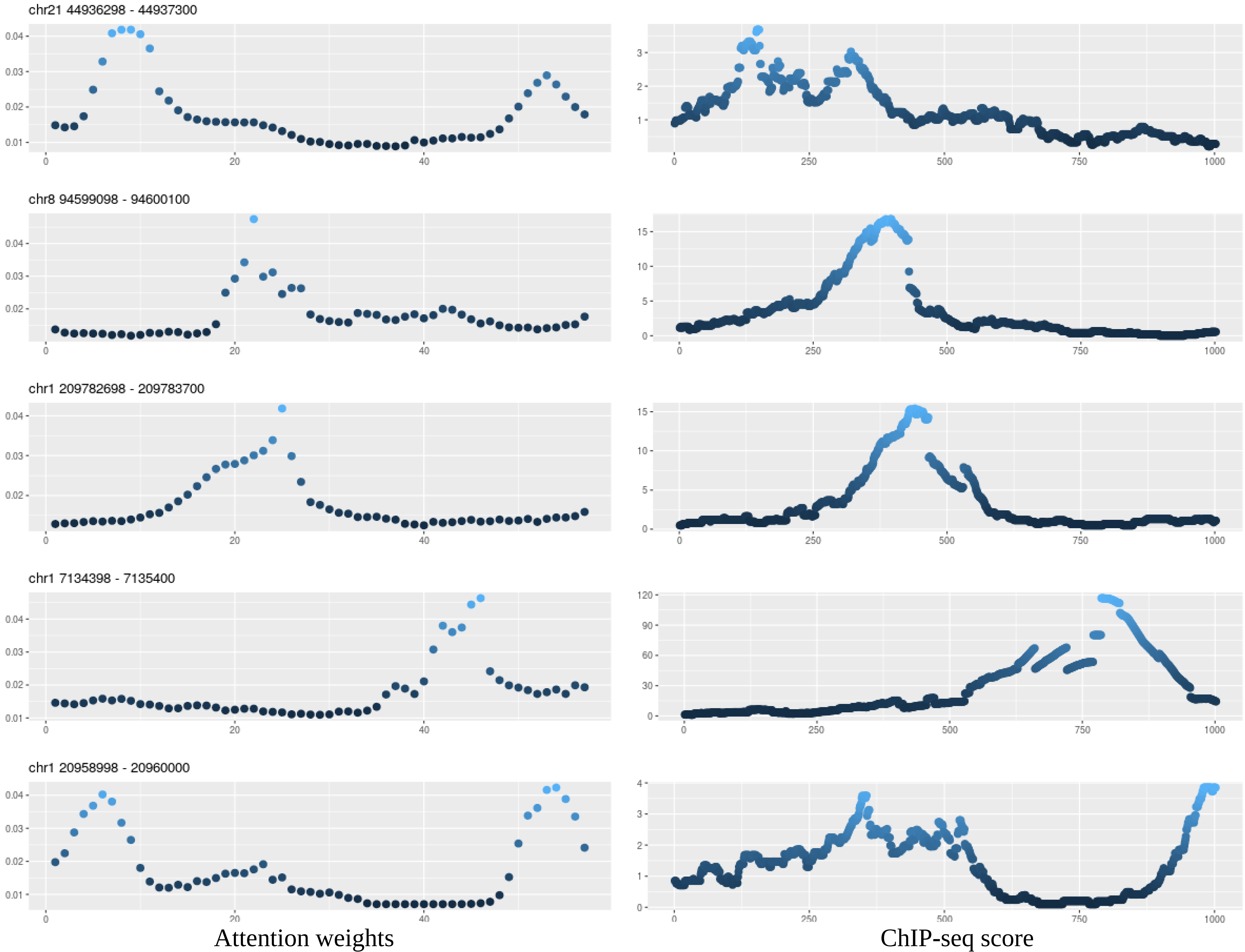

Single attention module

TAF1/liver

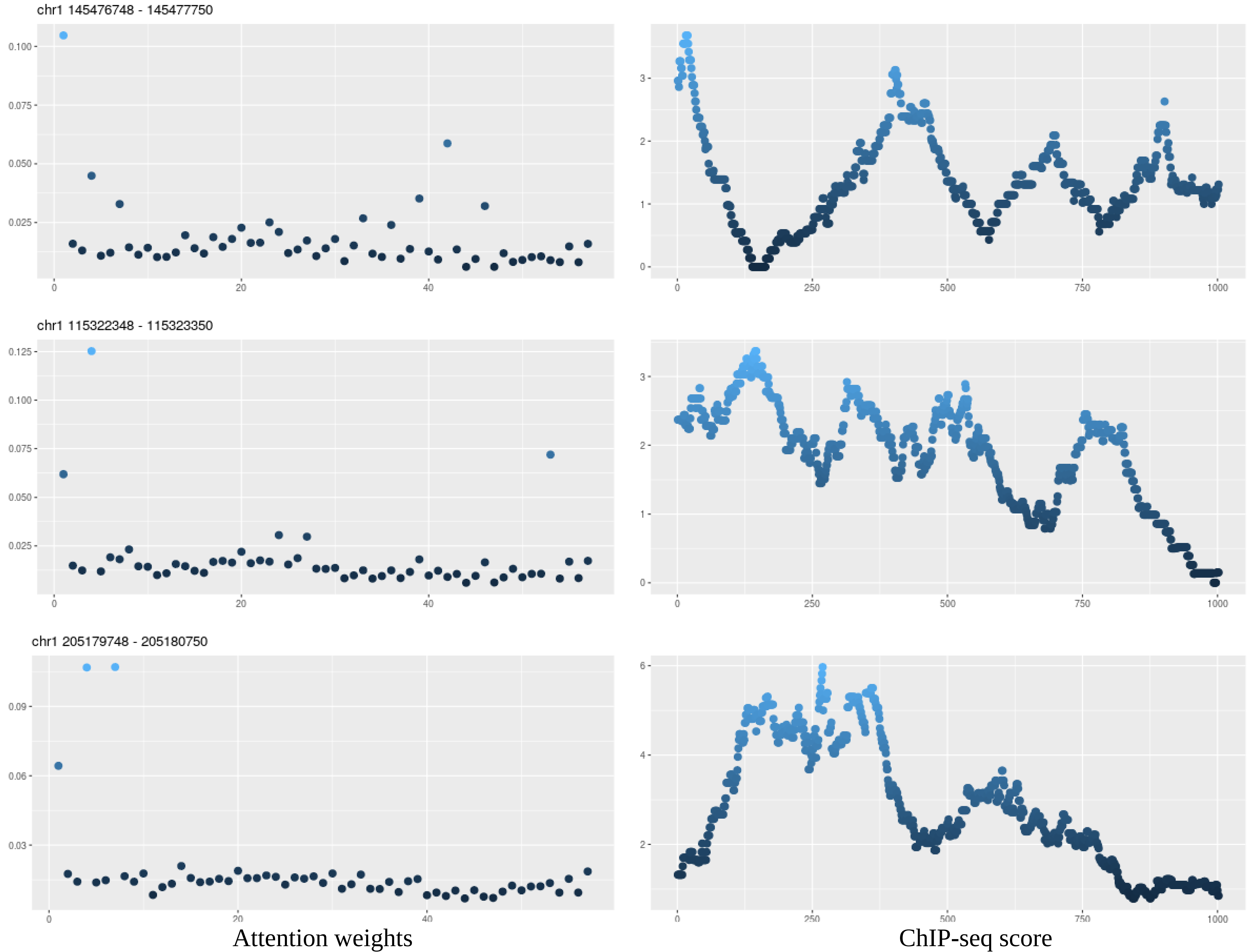

Pairwise attention module

CTCF/induced pluripotent stem cell

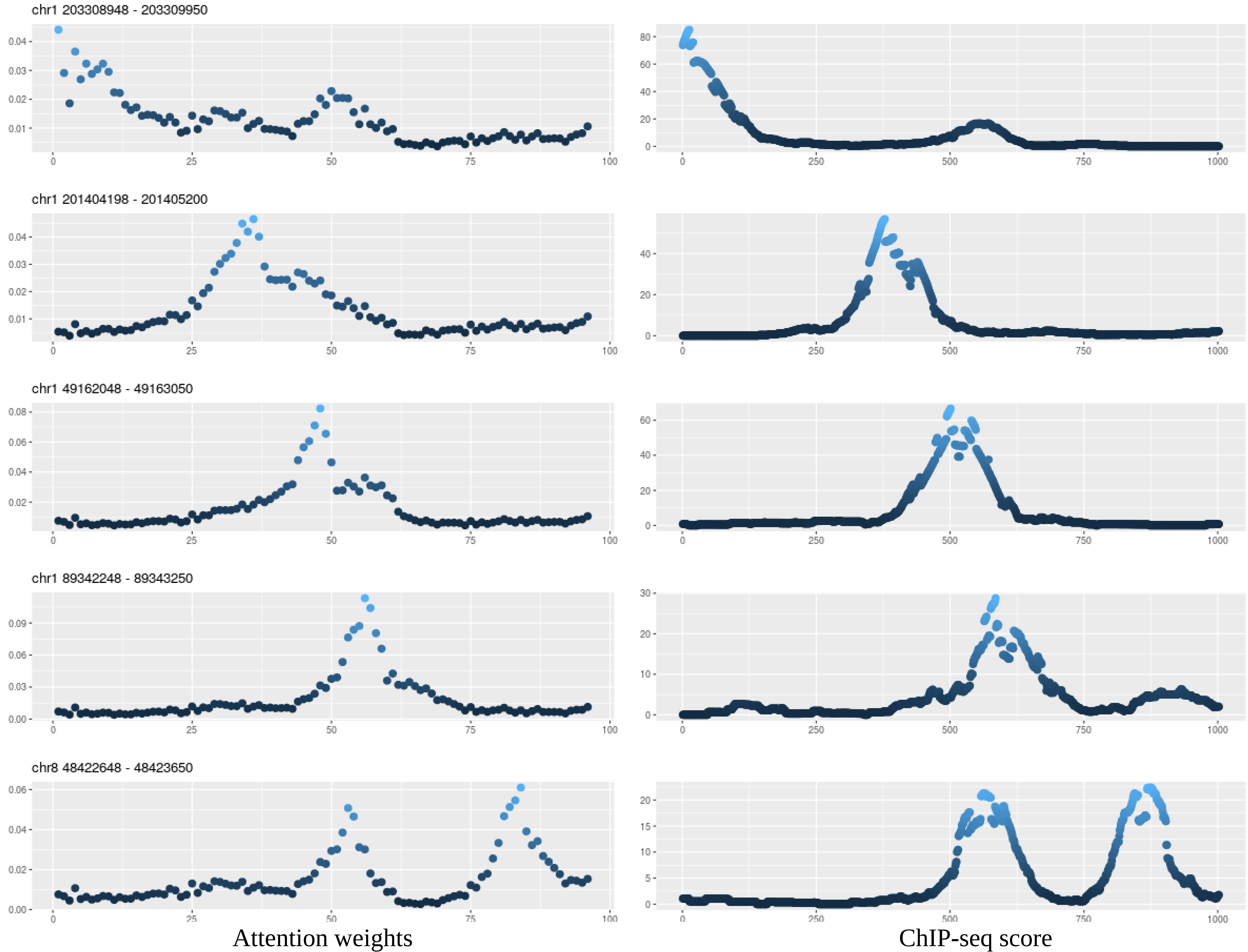

Pairwise attention module

CTCF/PC-3

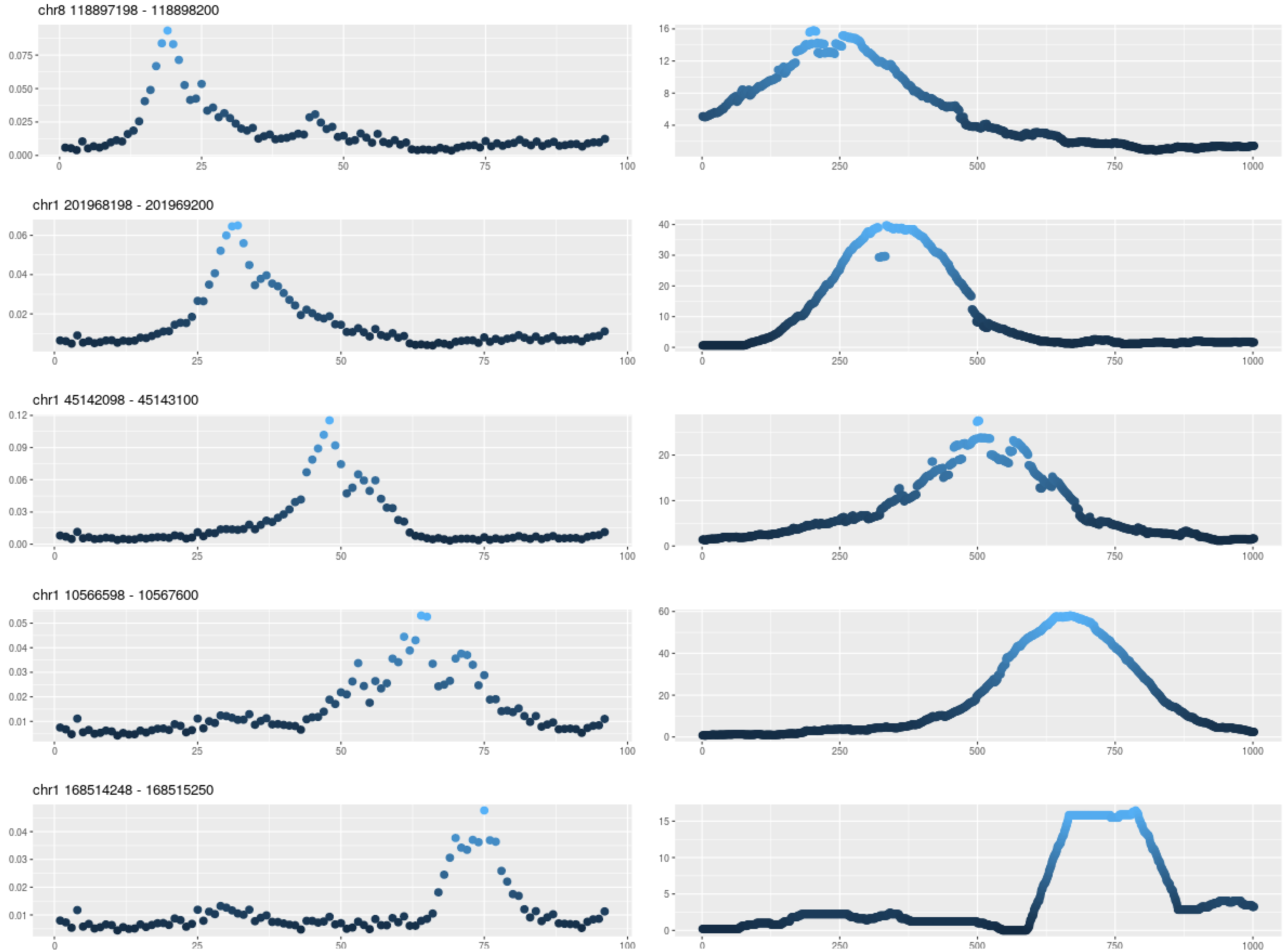

Attention weights ChIP-seq score

Pairwise attention module

E2F1/K562

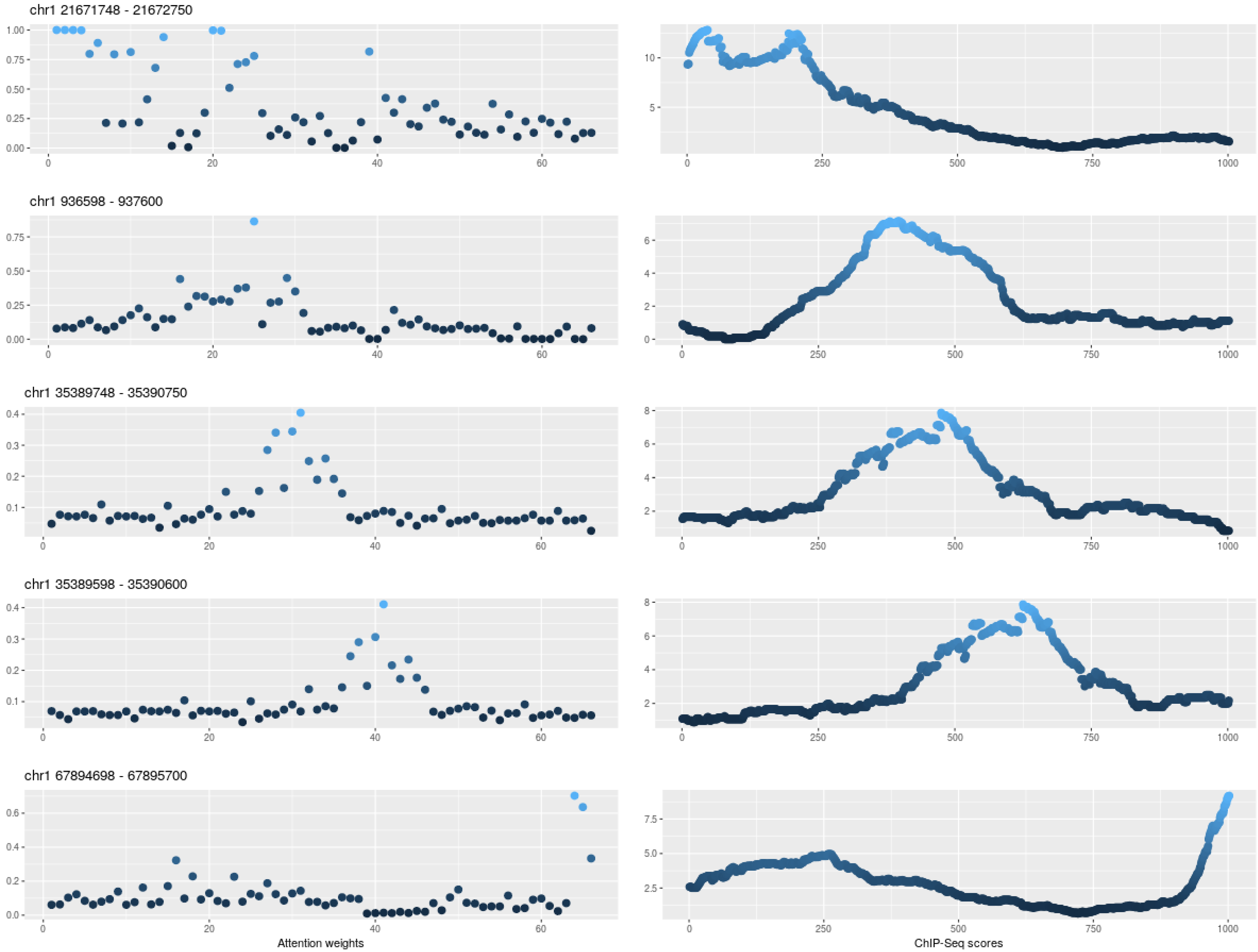

Pairwise attention module

EGR1/liver

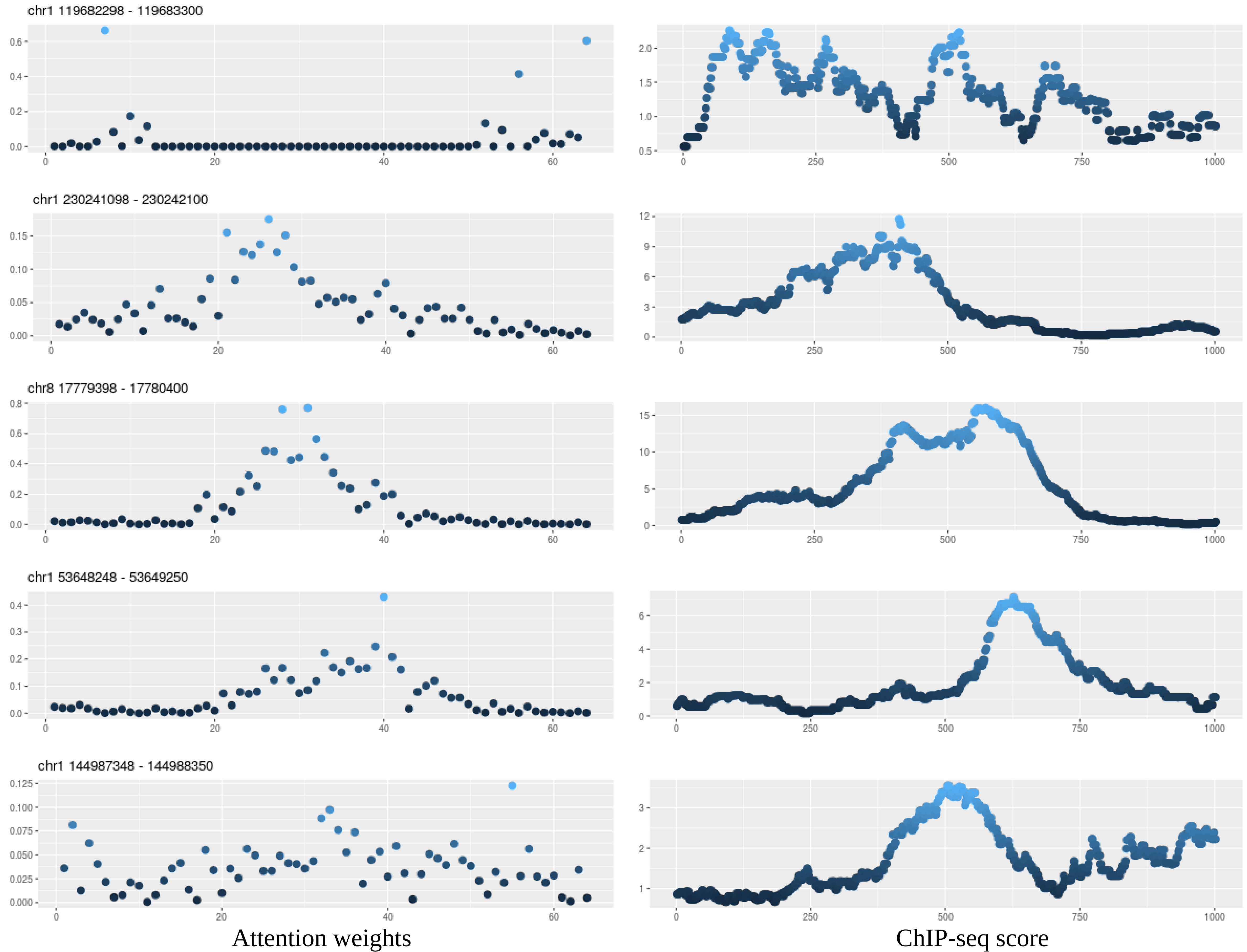

Pairwise attention module

FOXA1/liver

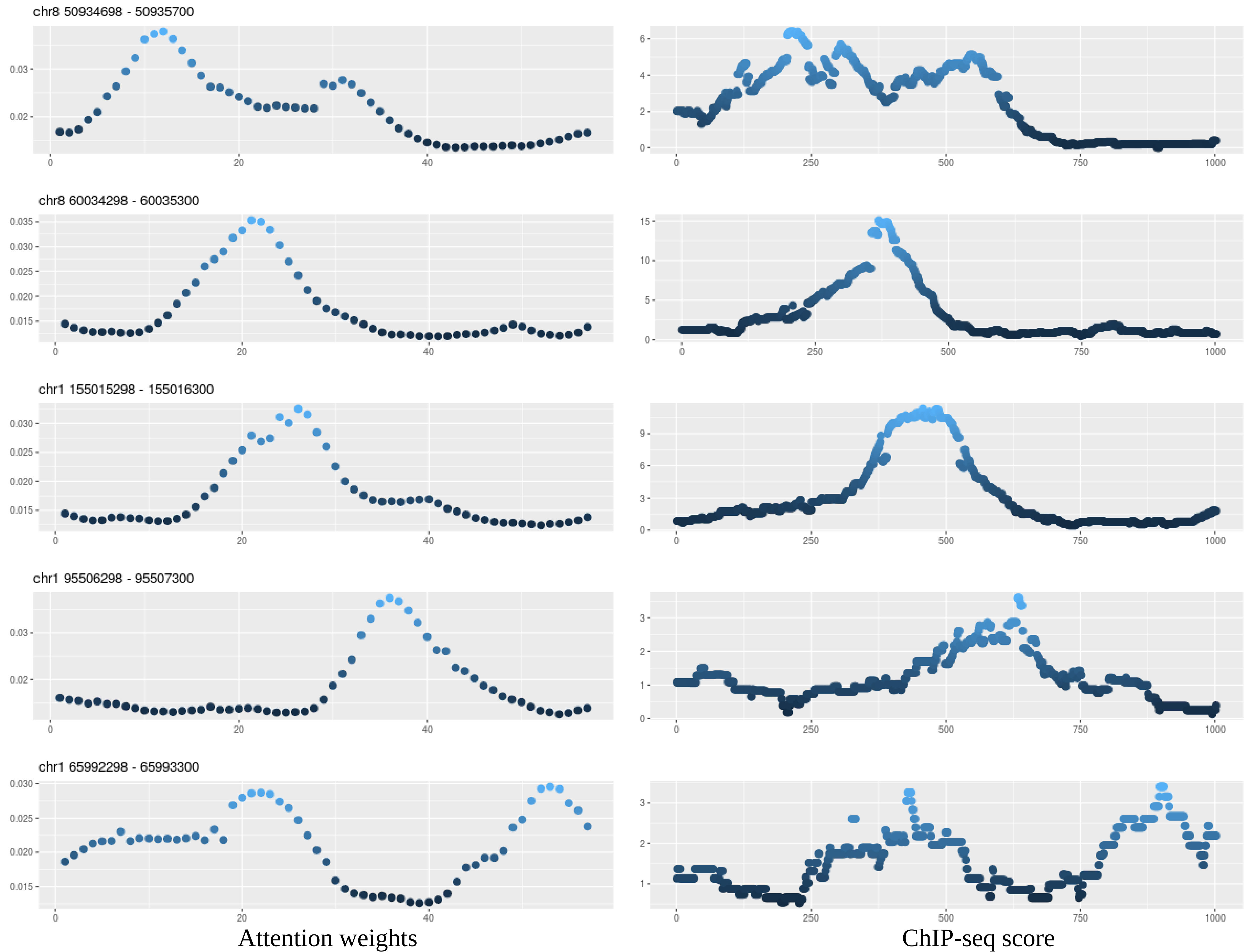

Pairwise attention module

FOXA2/liver

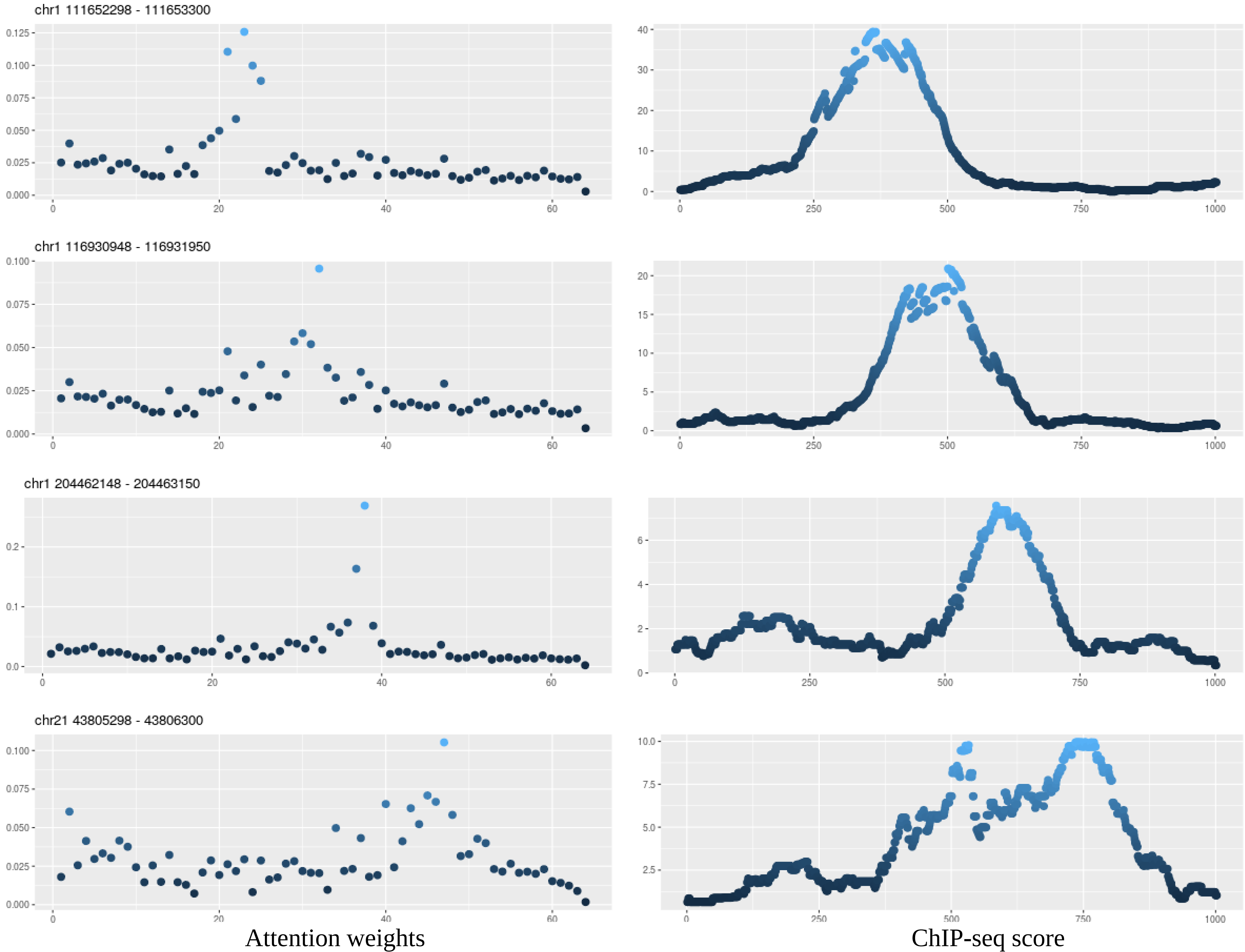

Pairwise attention module

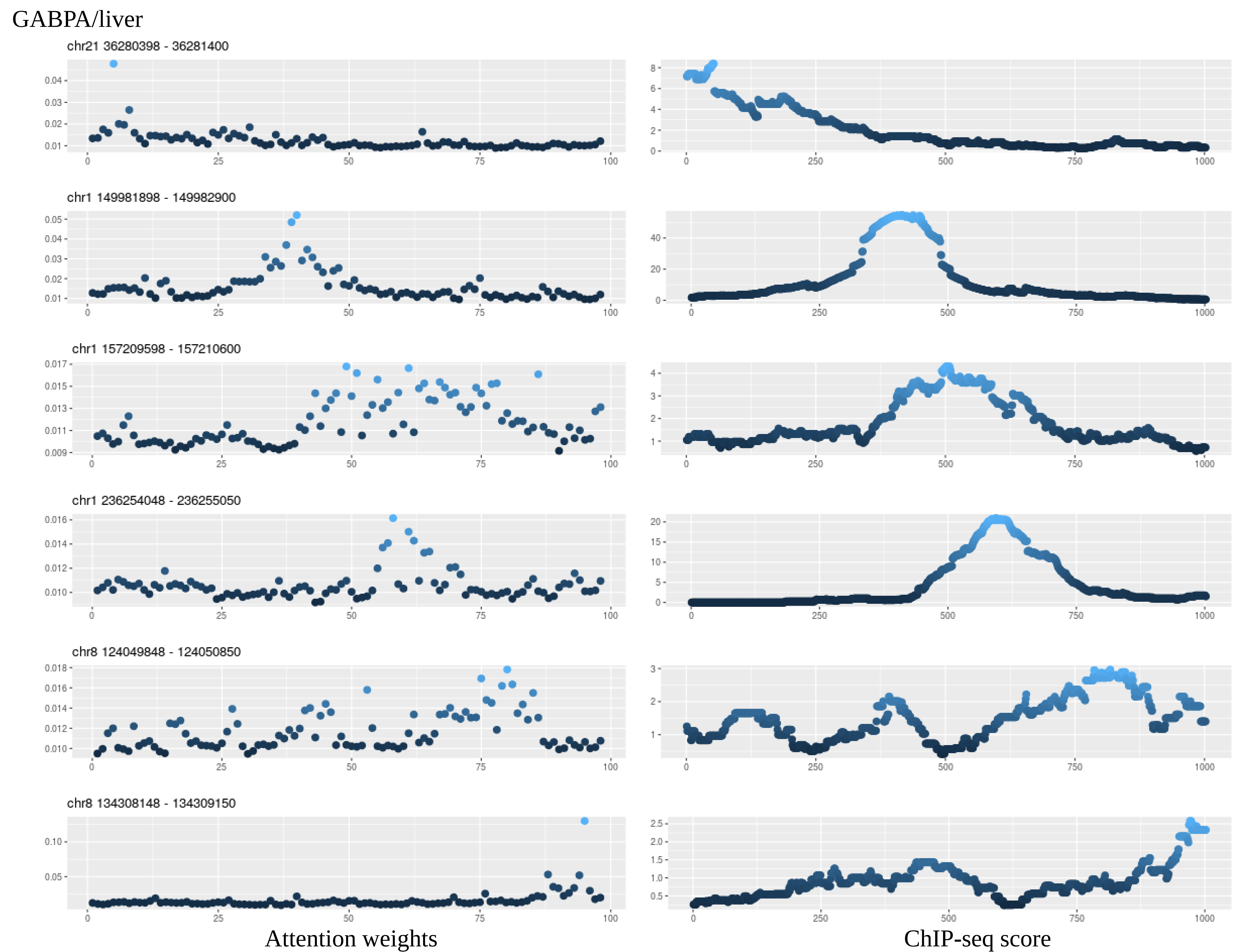

Pairwise attention module

HNF4A/liver

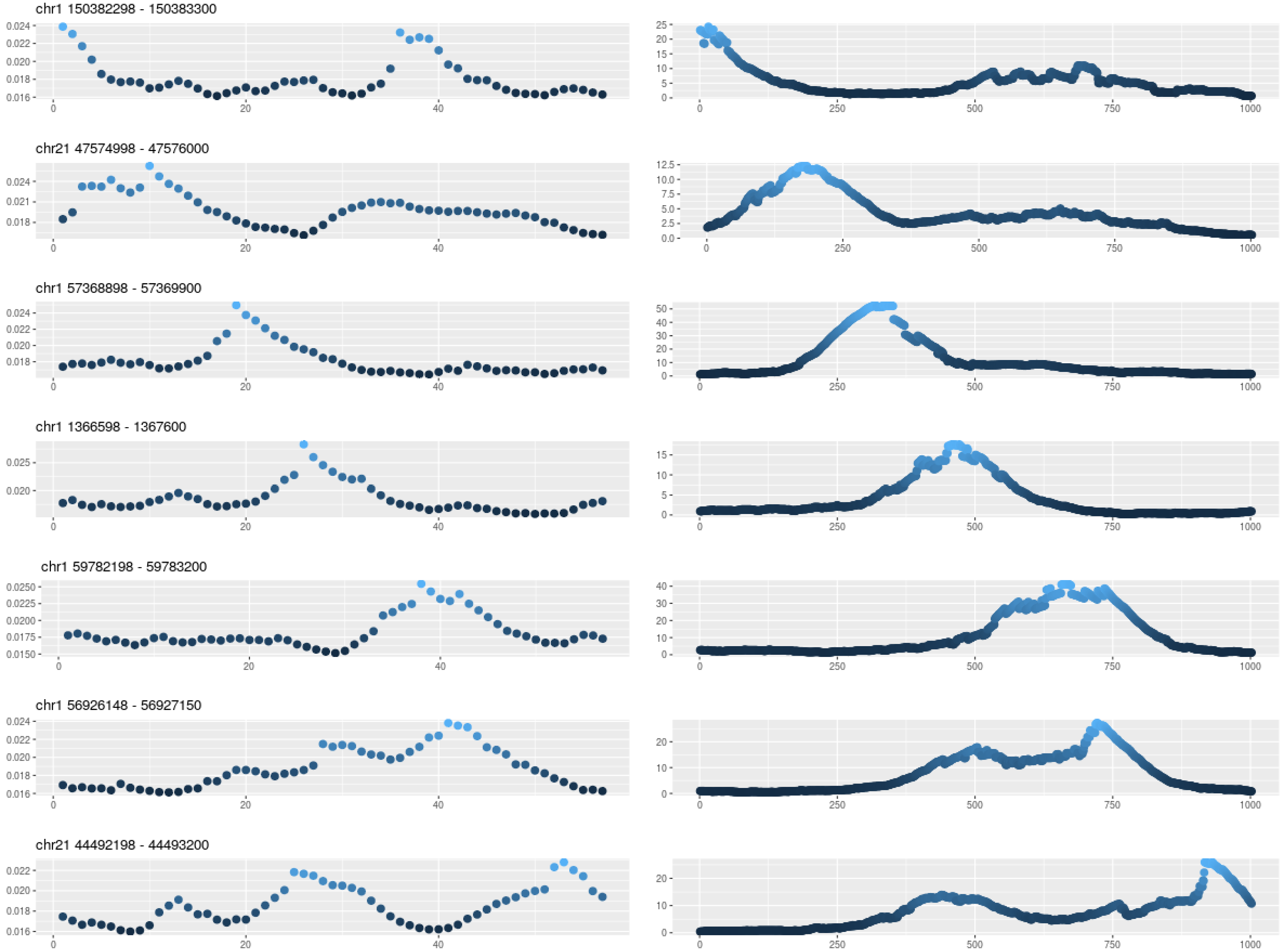

Attention weights ChIP-seq score

Pairwise attention module

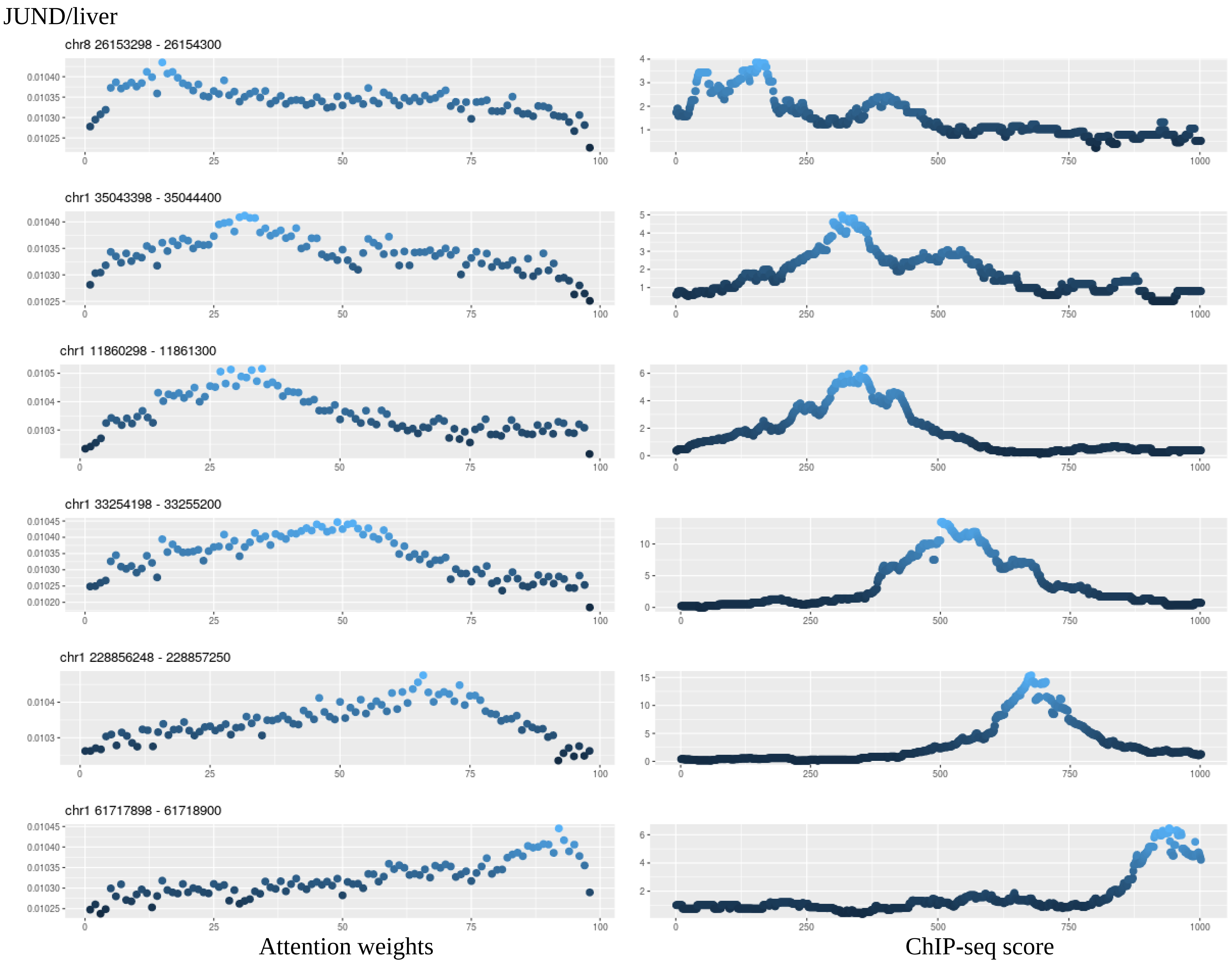

Pairwise attention module

MAX/liver

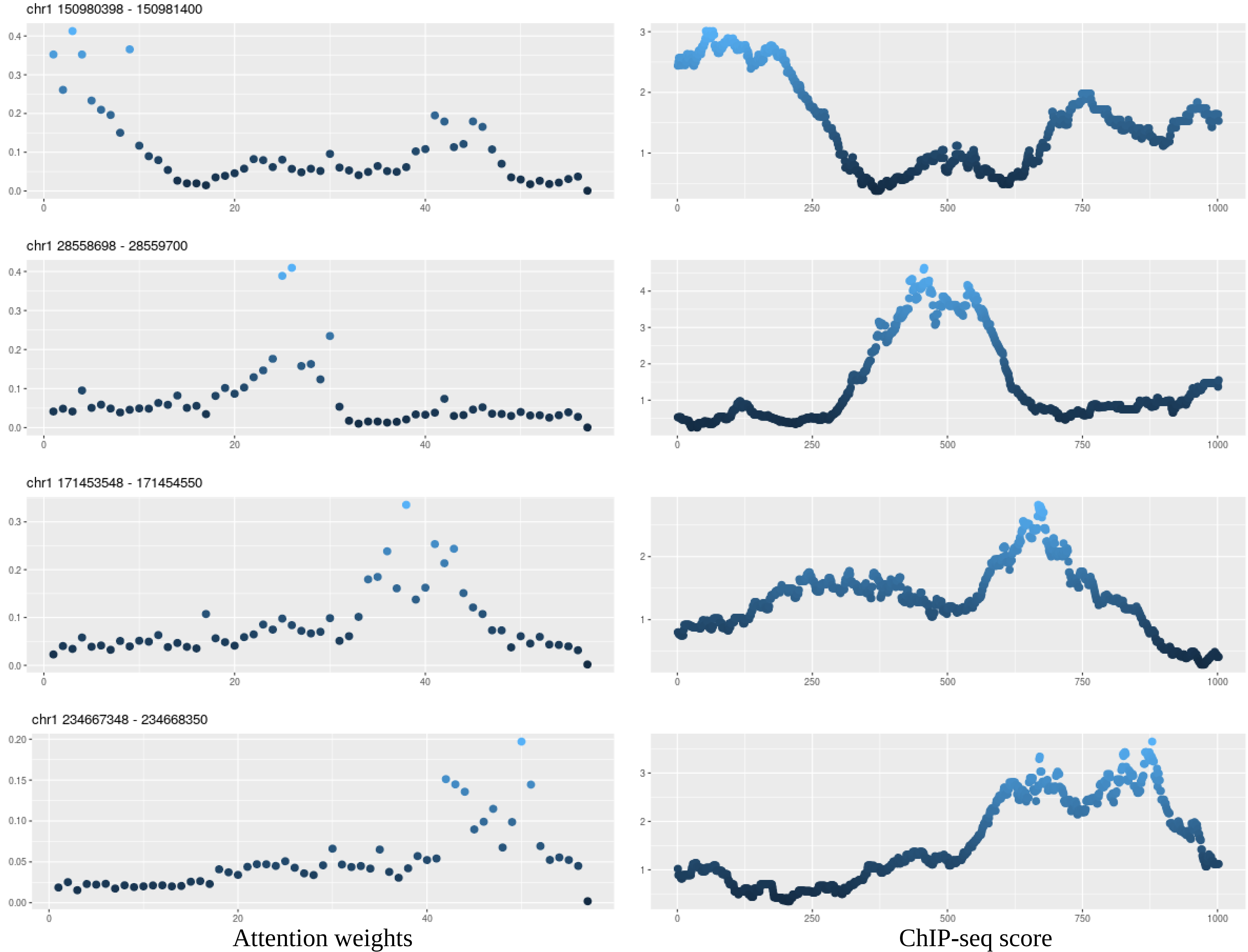

Pairwise attention module

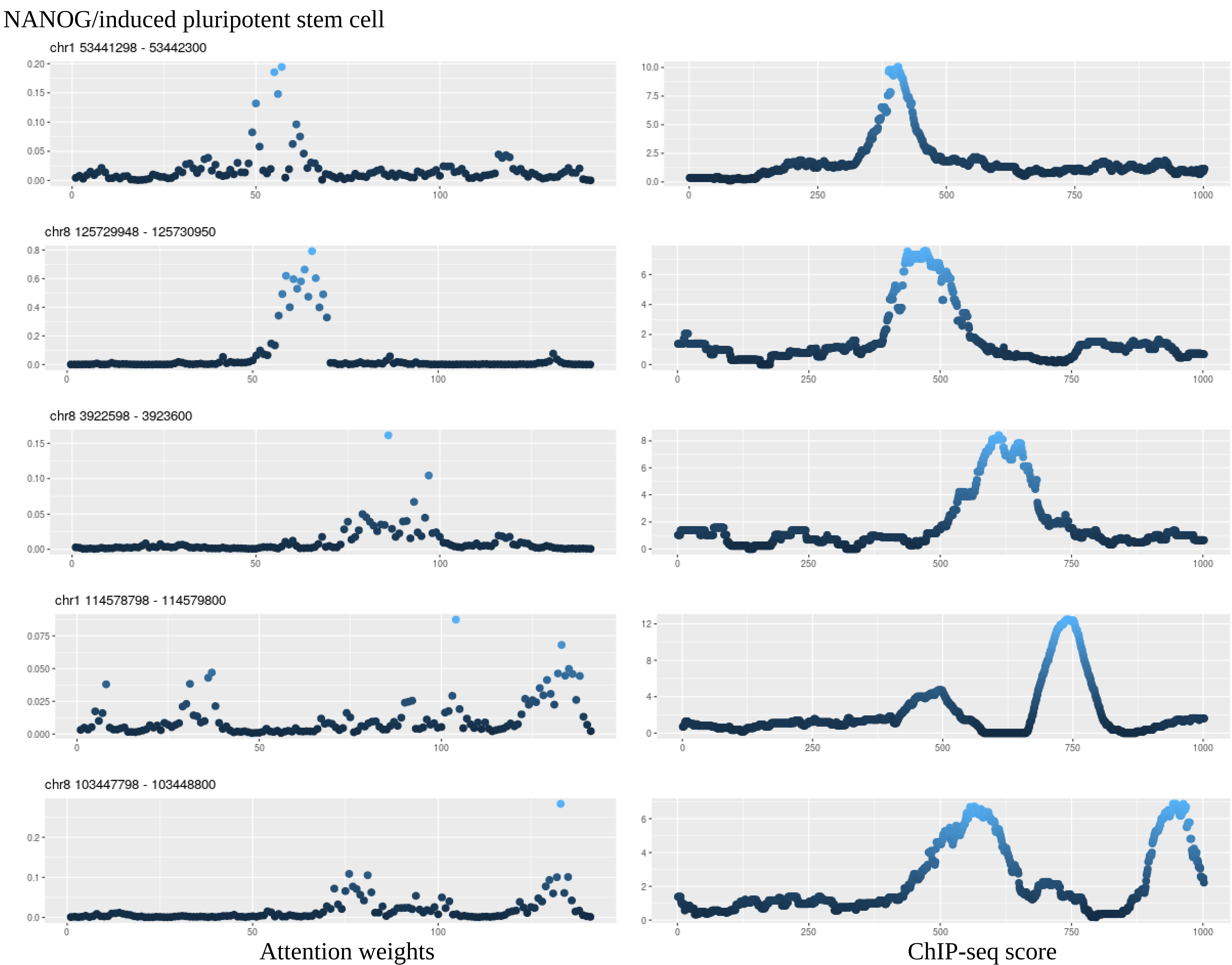

Pairwise attention module

REST/liver

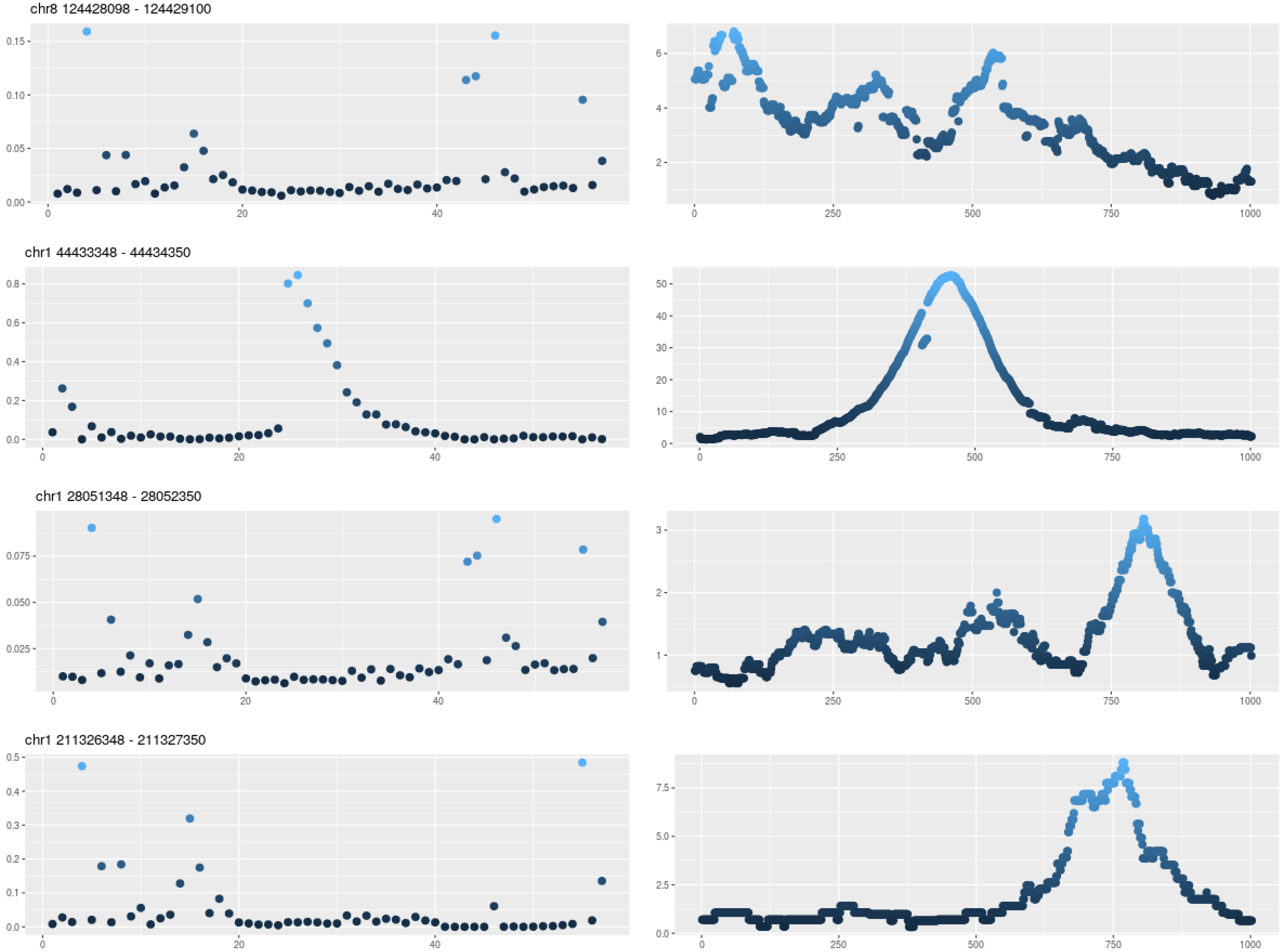

Attention weights ChIP-seq score

Pairwise attention module

TAF1/liver

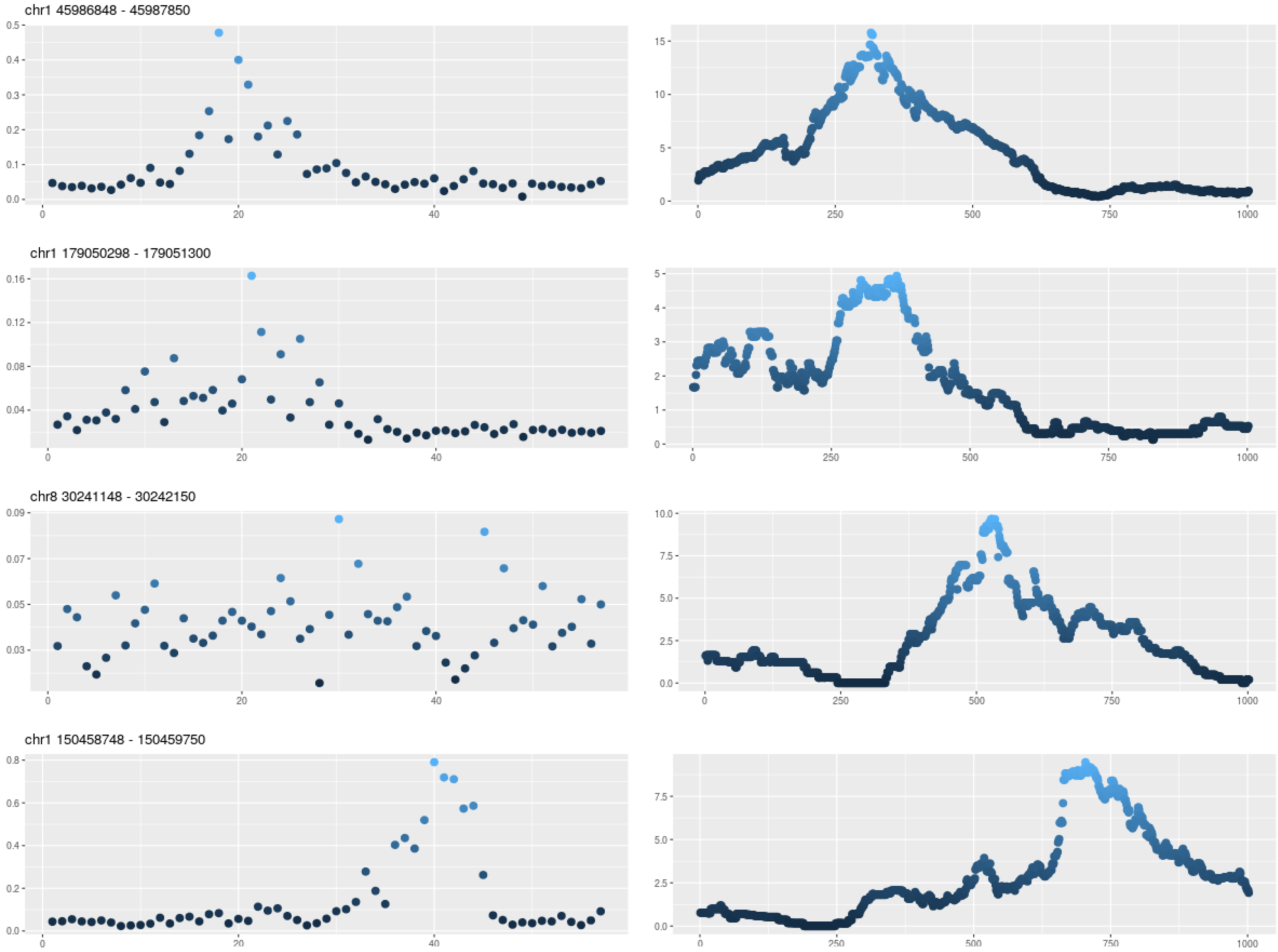

Attention weights ChIP-seq score
